# MicroRNAs regulate alveolar macrophage homeostasis and its function in lung fibrosis

**DOI:** 10.3389/fimmu.2025.1598306

**Published:** 2025-06-23

**Authors:** Nirmal Parajuli, Yi Yao, Namir Khalasawi, Congcong Yin, Qiong Zhang, Indra Adrianto, Aakash Hans, Li Zhou, Qing-Sheng Mi

**Affiliations:** ^1^ Center for Cutaneous Biology and Immunology Research, Department of Dermatology, Henry Ford Health, Detroit, MI, United States; ^2^ Immunology Research Program, Henry Ford Cancer Institute, Henry Ford Health, Detroit, MI, United States; ^3^ Center for Bioinformatics, Department of Public Health Sciences, Henry Ford Health, Detroit, MI, United States; ^4^ Department of Medicine, College of Human Medicine, Michigan State University, East Lansing, MI, United States; ^5^ Henry Ford Health + Michigan State University Health Sciences, Detroit, MI, United States; ^6^ Department of Internal Medicine, Henry Ford Health, Detroit, MI, United States

**Keywords:** alveolar macrophage, microRNA, pulmonary fibrosis, epigenetics, lung

## Abstract

**Introduction:**

Idiopathic pulmonary fibrosis is a progressive lung disease with a poor prognosis. Alveolar macrophages (AMs) are essential for maintaining lung homeostasis and play a significant role in the development of lung fibrosis. Tissue-Resident Alveolar Macrophages (TR-AMs), which originate from embryonic progenitors, can self-renew locally in a steady state, independent of hematopoiesis. During fibrogenesis, circulating monocytes rapidly migrate into the lungs and differentiate into monocyte-derived AMs (Mo-AMs). MicroRNAs (miRNAs), small non-coding RNAs, are critical for regulating gene expression. Our recent study found that the loss of miRNAs in embryonic progenitors significantly decreased the number of TR-AMs in late-stage embryos, indicating that miRNAs are necessary for TR-AM development. However, the role of miRNAs in the postnatal maintenance of TR-AMs and Mo-AMs, as well as their function in pulmonary fibrosis, remains unclear.

**Methods and Results:**

Here, we demonstrate that deleting miRNAs after birth severely disrupts TR-AM homeostasis and Mo-AM repopulation from the bone marrow following irradiation. The deficiency of miRNAs in TR-AMs and Mo-AMs was linked to diminished bleomycin-induced experimental lung fibrosis. Mechanistically, the absence of miRNAs increased TR-AM apoptosis under both normal and fibrotic conditions. RNA sequencing (RNA-seq) analysis revealed distinct transcriptomic and pathway changes in miRNA-deficient AM subgroups after lung injury. The integration of RNA-seq and miRNA array analyses identified miRNA-mRNA networks in TR-AMs and Mo-AMs in response to bleomycin injury. Ingenuity Pathway Analysis further predicted let-7a, miR-155, and miR-125 as unique upstream regulators of Mo-AM responses to lung fibrosis.

**Conclusions:**

Our findings suggest that miRNAs are key epigenetic mediators that differentially regulate the maintenance and function of TR-AMs and Mo-AMs in the pathogenesis of pulmonary fibrosis.

## Graphical Abstract

Simplified scheme of the model. Arrow-headed solid lines - biological processes; hammer-headed solid lines - inhibitory regulation; circle-headed solid lines - ineffective regulation; red arrows - upregulated gene expression; turquoise arrows - downregulated gene expression.
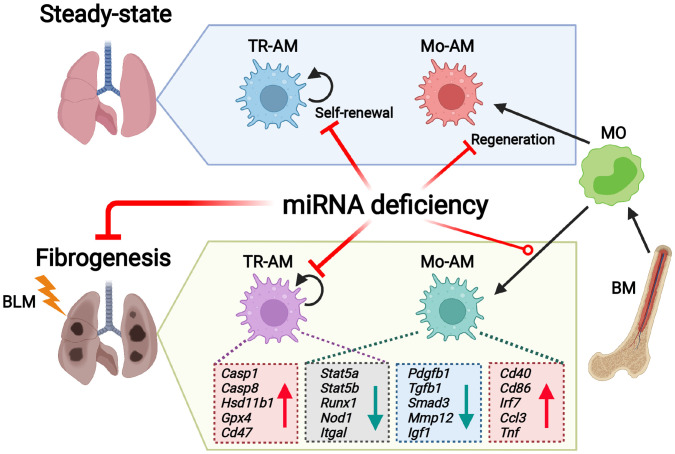

## 1 Introduction

Alveolar macrophages (AMs) are tissue-resident macrophages (TRMs) in the lungs that play a crucial role in maintaining airway homeostasis and are implicated in lung diseases, such as pulmonary fibrosis (PF) (1). Recent lineage tracing studies have revealed that TRMs originate from embryonic yolk sac (YS)-derived erythro-myeloid progenitors (EMPs) in distinct waves (2–4). In the first wave, YS EMPs differentiate into pre-macrophages (pMacs) that colonize all embryonic organs (5). These YS EMPs also migrate to the fetal liver (FL) starting at E12.5, where they expand and differentiate into pMacs and/or monocytes (MOs), which then travel to various tissues and mature into tissue-specific TRMs in the second wave (4). Fetal MOs begin to accumulate in the developing lung around E14.5, where they differentiate into F4/80^int^CD11b^int^ AM precursors (preAMs). These preAMs subsequently mature into CD11c^hi^Siglec-F^hi^CD11b^lo^ tissue-resident AMs (TR-AMs) in the alveolar space after birth (4, 6, 7). Under steady-state conditions, TR-AMs self-maintain in the lung throughout adulthood with minimal input from the bone marrow (BM) (4, 8). However, during lung injury, BM-derived circulating MOs are rapidly recruited into the lung, where they differentiate into MO-derived AMs (Mo-AMs), which exhibit distinct characteristics from TR-AMs (4, 8, 9).

Macrophage identity and homeostasis depend on the precise regulation of gene expression, which is controlled by various epigenetic mechanisms, including small non-coding miRNAs and histone modifications (10). While several key genes involved in alveolar macrophage (AM) development and homeostasis have been identified such as M-CSF (11), GM-CSF (7), TGF-β (12), and PPAR-γ (7, 12); much less is known about the epigenetic factors that govern embryonic AM development and postnatal maintenance. We recently reported that histone deacetylase 3 is essential for AM development, homeostasis, maturation, and regeneration from the BM (13). However, the role of other epigenetic regulators in AM development, maintenance, and function remains largely unknown.

MicroRNAs (miRNAs) are a class of evolutionarily conserved small non-coding RNAs (19–25 nucleotides) that negatively regulate gene expression and play important roles in development, tissue homeostasis, and disease progression (14, 15). The RNase III enzyme Dicer is crucial for processing miRNAs into their mature, functional forms, making its deletion a useful genetic tool for studying miRNA function. However, global deletion of Dicer (16), leads to developmental failure in mice, so conditional Dicer knockout (KO) models have been used to explore the roles of miRNAs in immune cell development and function (17–20). In our previous work using myeloid-lineage Csf1rCre-induced Dicer deletion mice, we observed that embryonic preAMs were nearly absent in Csf1r.DicerKO mice (21), highlighting the essential role of miRNAs in alveolar macrophage (AM) ontogeny. Nonetheless, it remains unclear whether miRNAs are necessary for AM maintenance postnatally and their involvement in lung diseases like pulmonary fibrosis (PF).

Idiopathic pulmonary fibrosis (IPF) is a chronic lung disease characterized by the aberrant activation of alveolar epithelial cells, accumulation of fibroblasts and myofibroblasts, and excessive production of extracellular matrix (22). Bleomycin (BLM)-induced pulmonary fibrosis (PF) is the most widely used experimental model of IPF (23). miRNAs are thought to play a significant role in the development of IPF, as altered miRNA expression patterns in both the blood and lungs of IPF patients are closely linked to disease onset (24–26). However, the specific roles of differentially expressed miRNAs in distinct cell types during IPF remain incompletely understood. Macrophages are involved in various aspects of fibrosis development, including the regulation of fibroblast proliferation, recruitment, and activation, as well as the direct modulation of extracellular matrix components and the secretion of profibrotic cytokines and growth factors (27). While the contribution of tissue-resident alveolar macrophages (TR-AMs) to fibrosis remains controversial (28, 29), there is increasing evidence supporting a profibrotic role for monocyte-derived macrophages (Mo-AMs) in experimental BLM-induced PF (28, 30, 31). However, whether miRNAs regulate the function of TR-AMs and Mo-AMs during PF development is still unclear.

In this study, we aim to investigate the role of miRNAs in the steady-state maintenance and bone marrow (BM)-derived repopulation of alveolar macrophages (AMs), as well as their function in the pathogenesis of experimental pulmonary fibrosis (PF). Using mice with CD11c-Cre- or Csf1r-Cre-mediated Dicer deletion and a BM chimeric model, we demonstrate that the absence of miRNAs results in severe defects in AM maintenance, maturation, and regeneration. In a bleomycin (BLM)-induced PF model, we show that miRNA loss, driven by CD11c-Cre-mediated Dicer deletion, induces significant changes in the transcriptomic profiles and signaling pathways of tissue-resident AMs (TR-AMs) and monocyte-derived AMs (Mo-AMs) during fibrogenesis, contributing to a reduction in the severity of lung fibrosis. Integrated RNA-seq and miRNA array analyses further reveal miRNA-mRNA regulatory networks in TR-AMs and Mo-AMs during PF development. Our findings highlight miRNAs as crucial epigenetic regulators of AM homeostasis and function in both health and disease.

## 2 Results

### 2.1 miRNAs are essential for the maintenance of mature TR-AMs in the lung

Most TRMs, TR-AMs, originate from the embryonic yolk sac and fetal live progenitors (2–4). After birth, immature TR-AMs differentiate into mature TR-AMs, which are characterized by increased expression of CD11c and Siglec F, alongside a decrease in CD11b expression (7, 8). Once mature, TR-AMs are capable of self-maintenance in the steady state throughout adult life, with minimal contribution from BM input (4, 8). To investigate whether miRNAs are essential for the steady-state maintenance of TR-AMs, we generated mice with a postnatal deficiency of miRNAs in AMs by crossing Dicer^fl/fl^ mice with CD11c^Cre^ transgenic mice, as CD11c is highly expressed in AMs after birth (7).

**Figure 1:**
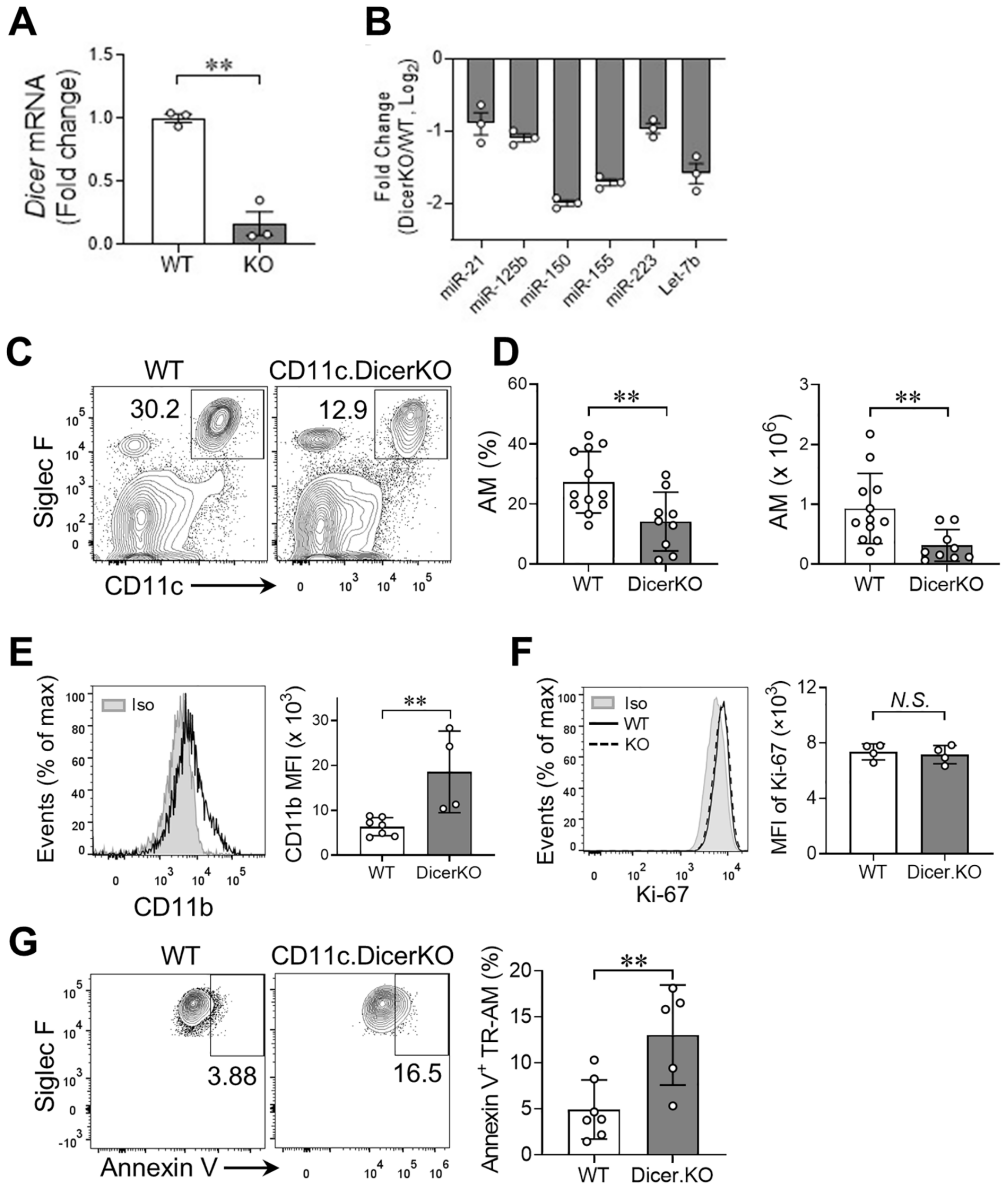
miRNAs are required for lung TR-AM homeostasis in the steady state. **(A)** mRNA expression of *Dicer* in TR-AMs from naïve *Dicer^fl/fl^* (WT) and *Cd11c^cre^Dicer^fl/fl^* (KO) adult mice analyzed by qRT-PCR. **(B)** Representative miRNA expression in TR-AMs from naïve WT and Dicer KO mice analyzed by qRT-PCR. **(C)** Representative flow cytometry plots: gated from CD45^+^ live cells of lungs from *Dicer^fl/fl^* (WT) and *Cd11c^Cre^Dicer^fl/fl^* (KO) adult mice. **(D)** Frequencies and absolute numbers of TR-AMs (CD11c^hi^Siglec-F^hi^) as in **(A)** n = 12 *Dicer^fl/fl^*, n = 9 *Cd11c^Cre^Dicer^fl/fl^*. **(E)** Histogram plot for CD11b expression of lung TR-AMs from n = 10 *Dicer^fl/fl^*, n = 9 *Cd11c^Cre^Dicer^fl/fl^* adult mice. The mean fluorescence intensity (MFI) of CD11b is shown on the right. **(F)** Histogram plot for Ki-67 expression of lung TR-AMs from n = 7 *Dicer^fl/fl^*, n = 5 *Cd11c^Cre^Dicer^fl/fl^* adult mice. Rat IgG2a served as an isotype (Iso) control. The MFI of Ki-67 is shown on the right. **(G)** Representative flow cytometry plots for annexin V staining of TR-AMs. Frequencies of apoptotic (annexin V^+^) cells within TR-AMs are shown on the right. n = 7 *Dicer^fl/fl^*, n = 5 *Cd11c^Cre^Dicer^fl/fl^*. Each dot represents one mouse and bars represent mean ± SD of biologically independent samples in each panel. All *P* values were obtained by the Student’s two-tailed unpaired *t* test. **P < 0.01. N*.S.*, not significant.

We observed an over 80% reduction of *Dicer* mRNA levels in TR-AMs from naïve *CD11c^Cre^Dicer^fl/fl^* KO adult mice compared to *Dicer^fl/fl^* wild-type (WT) controls (**Figure 1A**). Meanwhile, Dicer-deficient TR-AMs expressed lower levels of miRNAs that have been reported to be implicated in macrophage activation, polarization, and function, such as miR-21, miR-150, miR-155, and miR-223 (**Figure 1B**), which further validates the efficacy of miRNA depletion in the DicerKO mice as reported previously (19). We first analyzed the frequency and number of TR-AMs (CD45^+^Siglec-F^hi^CD11c^hi^) in the lung tissue compartments of naïve *Dicer^fl/fl^* and *CD11c^Cre^Dicer^fl/fl^* adult mice, and found that they were both significantly decreased in the DicerKO mice compared to the WT mice (**Figures 1C, D**, gating strategy shown in **Supplementary Figure 2A**). We next measured the maturation marker CD11b of TR-AMs and found that there was higher expression of CD11b in DicerKO AMs compared to WT AMs (**Figure 1E**), suggesting a less-mature status of TR-AMs in the absence of miRNAs. These results imply that miRNAs are required for fully mature TR-AM postnatal maintenance at steady state.

The steady-state AM network can be maintained by a slow local proliferation of AMs rather than by BM precursors (8). To determine whether a reduced number of lung TR-AMs in miRNA-deficient mice resulted from impaired proliferation capacity of TR-AMs, we measured the expression of Ki-67 protein, a cell proliferation marker, in TR-AMs from *Dicer^fl/fl^* WT and *CD11c^Cre^Dicer^fl/fl^* KO mice. We found comparable expression of Ki-67 protein in TR-AMs of both groups (**Figure 1F**), suggesting that lack of miRNAs did not affect TR-AM proliferation capacity. We next determined whether loss of miRNAs affects cell survival that may lead to deficient TR-AMs in *CD11c^Cre^Dicer^fl/fl^* mice. Annexin V staining showed increased frequency of annexin V^+^ apoptotic TR-AMs in DicerKO mice compared to WT mice (**Figures 1G**). Taken together, these results imply that miRNAs are required for lung TR-AM steady-state maintenance, possibly through regulation of cell survival rather than cell proliferation of TR-AMs.

### 2.2 miRNAs are required for Mo-AM repopulation from the BM after irradiation

**Figure 2:**
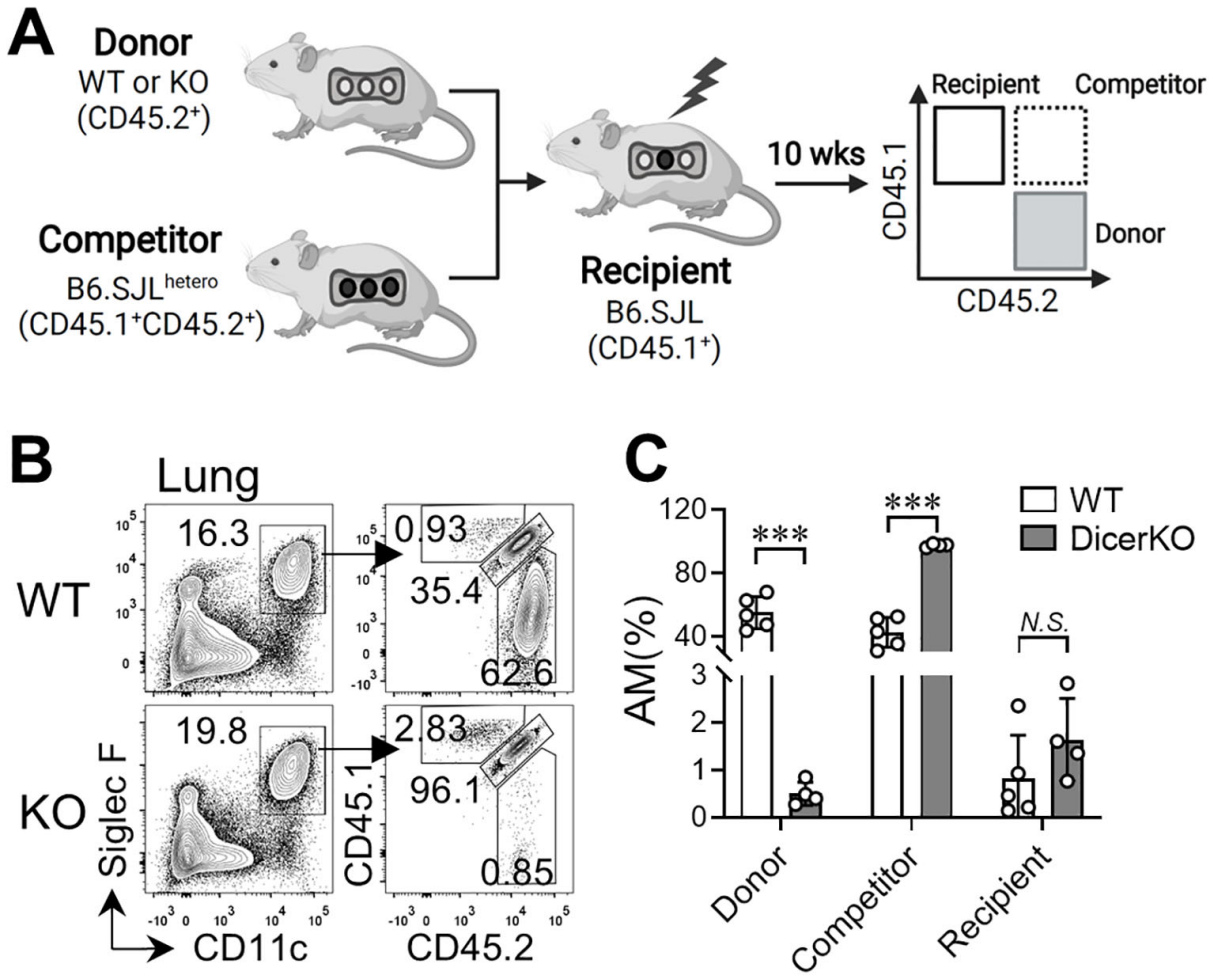
miRNAs are required for AM regeneration from bone marrow in the steady state. Bone marrow (BM) cells from *Dicer^fl/fl^* and *Csf1r^icre^Dicer^fl/fl^* mice (CD45.2^+^) were co-transferred with competitor BM cells from B6.SJL^hetero^ mice (CD45.1^+^CD45.2^+^) into lethally-irradiated B6.SJL mice (CD45.1^+^). Lungs were harvested from recipient mice 10 wks after reconstitution. **(A)** Schematic representation of the BM chimeric mouse working models. **(B)** Flow cytometry of CD45.1 and CD45.2 expression on CD11c^hi^Siglec-F^hi^ lung AMs from n = 5 *WT: Dicer^fl/fl^*, n = 4 WT: *Csf1r^icre^Dicer^fl/fl^* mice. Bars represent mean ± SD of biologically independent samples. All *P* values were obtained by the Student’s two-tailed unpaired *t* test. ***P < 0.001. N*.S.*, not significant.

Self-renewal of TR-AMs is independent of circulating precursors at steady state (9). However, under certain stress or inflammatory conditions, BM-derived monocytes can differentiate into Mo-AMs and repopulate the AM niche (9, 32). To determine whether the repopulation of Mo-AMs depends on miRNAs, we applied an established BM chimeric mouse model (13) by co-transferring BM cells from *Dicer^fl/fl^* WT or *Csf1r^iCre^Dicer^fl/fl^* KO donors (CD45.2^+^) with competitor BM cells from B6.SJL^hetero^ mice (CD45.1^+^CD45.2^+^) into the irradiated B6.SJL (CD45.1^+^) recipient mice (**Figure 2A**). In the BM chimeric mice, very few (<3%) CD45.1^+^cells within the total AM population existed in the lung, representing leftover TR-AMs from the recipient mice after irradiation (**Figures 2B, C**). Meanwhile, the AM niche was almost equally occupied by Mo-AMs of WT donor and competitor origins. However, when the BM cells from DicerKO donors were co-transferred with competitor cells, the AM niche was nearly completely reconstituted by Mo-AMs of competitor origin. These findings suggest that miRNAs are required for the repopulation of Mo-AMs from adult BM following lethal irradiation.

### 2.3 Establishment of BLM-induced lung fibrosis model using C57BL/6 mice

Emerging evidence reveals the important role of AMs in the pathogenesis of experimental PF (29, 31). To delineate the mechanisms regulating AM functions during fibrogenesis, we applied a BLM-induced PF mouse model, which has been widely used for experimental PF (28, 29, 33, 34). When C57BL/6 mice were treated with various doses of BLM via intratracheal instillation (**Supplementary Figure 3A**), we observed a dramatic decrease in the survival rate of mice treated with higher doses of BLM (1.5 and 3.0 U/kg) starting from 10 days after treatment, while the vehicle control and lower doses of BLM (0.5 and 1.0 U/kg) had no effect on mouse survival up to 14 days after treatment (**Supplementary Figure 3B**). These results are in line with previous reports showing less tolerance of C57BL/6 mice to BLM treatment (35). Meanwhile, fibrogenesis was assessed 14 days post-treatment by measuring lung tissue hydroxyproline levels, which are associated with collagen as an indicator of the severity of fibrosis (36, 37). As shown in **Supplementary Figure 3C**, a mild but significant increase in hydroxyproline content was detected in the lungs of mice treated with as low as 0.5 U/kg of BLM compared to the vehicle-treated control mice, while a peak of lung hydroxyproline content was observed in mice treated with 1.0 U/kg of BLM, along with a slight decrease in hydroxyproline levels for the mice treated with 1.5 and 3.0 U/kg of BLM. These findings suggest that a low dose of BLM is sufficient to induce PF in C57BL/6 mice and we chose 1.0 U/kg of BLM as the working concentration for the experimental PF mouse model.

**Figure 3:**
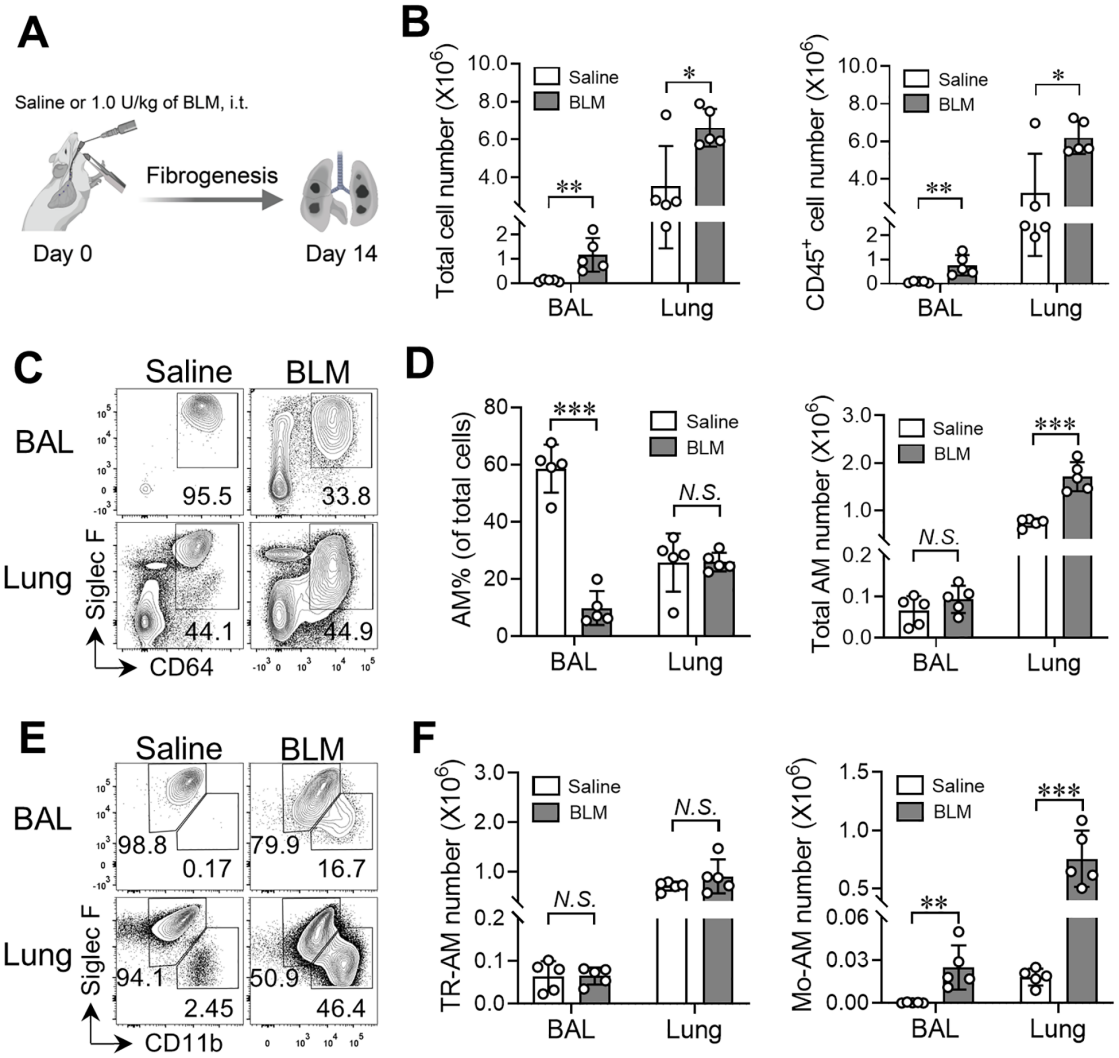
Emergence of Mo-AMs during bleomycin-induced pulmonary fibrosis. **(A)** Schematic representation of the bleomycin (BLM)-induced lung fibrosis model. **(B)** The numbers of total cells and CD45^+^ cells in bronchoalveolar lavage (BAL) and lungs from adult C57BL/6 mice treated with saline or BLM. **(C)** Representative flow cytometry plots for CD64 and Siglec-F expression within CD45^+^ live cells in BAL and lungs. **(D)** The frequencies and numbers of total AM cells (CD64^+^Siglec-F^+^) in BAL and lungs as in **(C, E)** Representative flow cytometry plots for CD11b and Siglec-F expression within CD64^+^Siglec-F^+^ total AM cells in BAL and lungs from adult C57BL/6 mice treated with saline or BLM. **(F)** The numbers of TR-AMs (CD11b^lo^Siglec-F^hi^) and Mo-AMs (CD11b^hi^Siglec-F^lo^) in BAL and lungs as in **(E)** Bars represent mean ± SD of biologically-independent samples. All *P* values were obtained by the Student’s two-tailed unpaired *t* test. *P < 0.05. **P < 0.01. *** *P* < 0.001.

To evaluate the cellular components in the fibrotic lungs, we treated WT mice (C57BL/6 background) with 1.0 U/kg of BLM or saline alone and analyzed the cellular compartments in both bronchoalveolar lavage (BAL) and lungs of mice on day 14 after treatment (**Figure 3A**). We found the numbers of total cells and immune cells (CD45^+^) were dramatically elevated in the BAL and lungs after BLM injury compared to the saline-treated control group (**Figure 3B**). Using flow cytometry, we further defined the total AM population based on expression of CD64 and Siglec F, and were able to distinguish TR-AMs (SiglecF^hi^CD11b^lo^) and Mo-AMs (SiglecF^lo^CD11b^hi^) within the CD64^+^SiglecF^+/lo^ AM population in BLM- or saline-treated WT mice, consistent with previous studies (**Figures 3C, E**, gating shown in **Supplementary Figure 2B**) (28, 31). Although CD11c was expressed by both TR-AMs and Mo-AMs at comparable levels, CD64 served as a better marker to separate the total AM population from other cell lineages (**Supplementary Figure 2B**). As expected, most cells residing in the airspace were AMs in saline-treated mice, while the frequency of AMs within total BAL cells was substantially decreased in BLM-treated mice, which suggests vigorous infiltration of other immune cell types into the airways following BLM treatment (**Figure 3D**). Meanwhile, we found that the numbers of total AMs were significantly increased in both BAL and lungs of BLM-treated WT mice compared to saline-treated mice (**Figure 3D**). Specifically, the numbers of TR-AMs were remained unchanged in the lung tissues of mice given BLM (**Figures 3E, F**). Moreover, the numbers of Mo-AMs were robustly elevated in the BAL and lungs of BLM-treated mice, which is consistent with other reports showing the emergence of Mo-AMs in both airspace and tissue compartments after BLM injury (28, 31). These results further support our BLM-PF mouse model as a valid tool to investigate AM subset homeostasis and function during experimental PF.

### 2.4 Reduced severity of lung fibrosis in CD11c-Cre-Dicer^fl/fl^ mice after BLM injury

**Figure 4:**
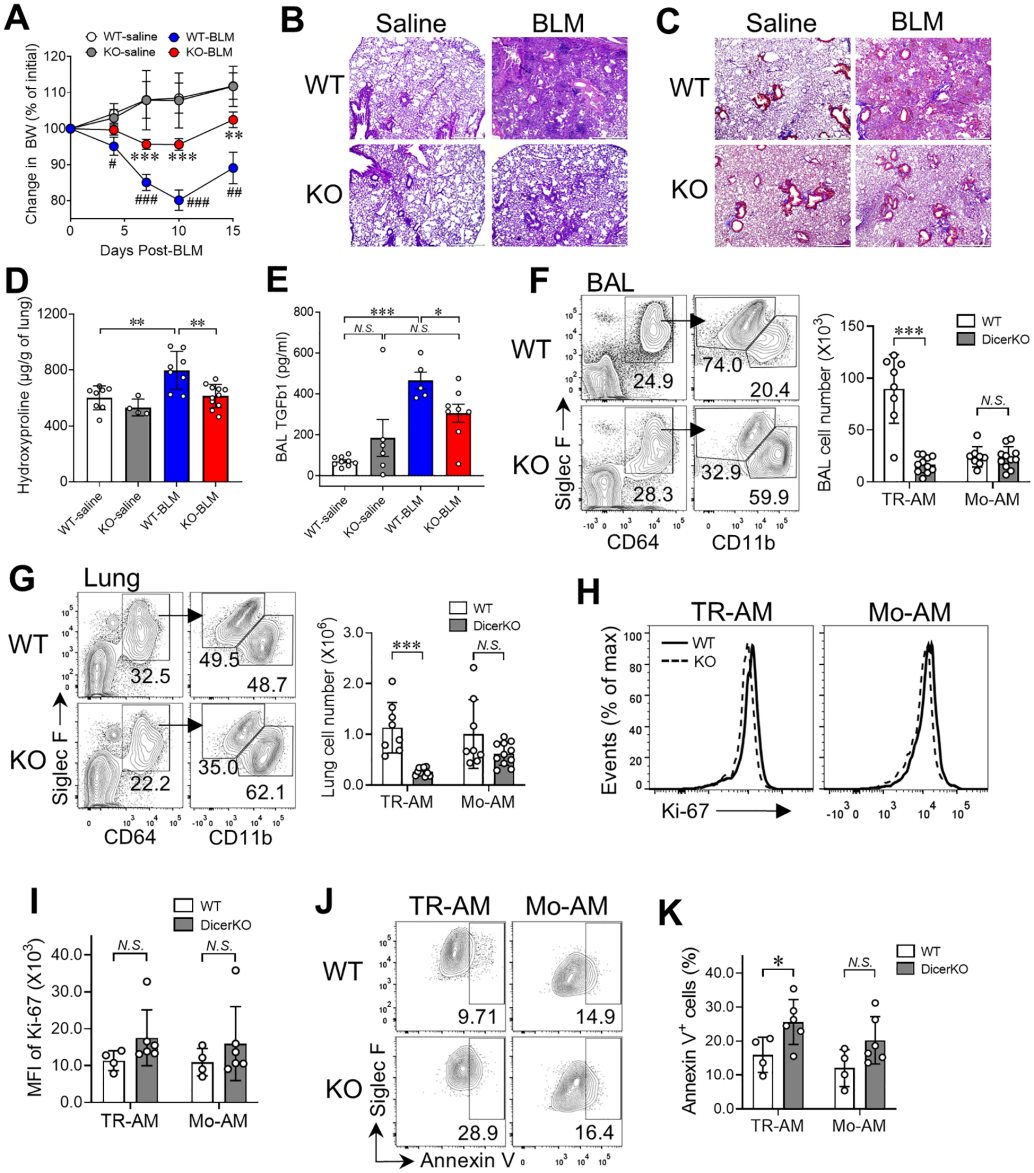
miRNA deficiency in AMs prevents bleomycin-induced pulmonary fibrosis. *Dicer^fl/fl^* (WT) and *Cd11c^cre^Dicer^fl/fl^* (KO) mice were intratracheally administered with saline or BLM and were monitored for fibrosis progression for 15 days after treatment. **(A)** Mouse body weights were measured on days 0, 4, 7, 10, and 15 after treatment. Plots showing normalized body weights at each time point compared to day 0. #, comparisons between BLM- and saline-treated WT mice; *, comparisons between WT and KO mice given BLM. **(B-K)** Mouse BAL and lungs were collected to assess fibrosis at day 15. H&E stain **(B)** and trichrome stain **(C)** on paraffin-embedded lung sections. Scale bars: 500 µm. **(D)** Hydroxyproline levels in the lungs. **(E)** TGFβ1 levels in BAL. **(F-G)** Representative flow cytometry plots for TR-AMs (CD64^+^CD11b^lo^Siglec-F^hi^) and Mo-AMs (CD64^+^CD11b^hi^Siglec-F^lo^) in the BAL **(F)** and lungs **(G)**. The numbers of TR-AMs and Mo-AMs in the BAL and lungs are on the right. **(H)** Histogram plots of Ki-67 expression of lung TR-AMs and Mo-AMs in the lungs. **(I)** The MFI of Ki-67 expression as in **(H, J)** Representative flow cytometry plots for annexin V staining for TR-AMs and Mo-AMs. **(K)** Frequencies of apoptotic (annexin V^+^) cells within lung TR-AMs and Mo-AMs. Each dot represents one mouse and bars represent mean ± SD of biologically independent samples in each panel. All *P* values were obtained by the Student’s two-tailed unpaired *t* test. **P < 0.01; ***P < 0.001. N*.S.*, not significant.

CD11c-Cre-driven conditional gene deletion mice have been commonly used to study the behavior and function of CD11c-expressing cells including AMs and dendritic cells (DCs) in the normal and pathological lung (7, 38, 39). To determine whether loss of miRNAs influences maintenance and function of TR-AMs and Mo-AMs during fibrogenesis, we intratracheally instilled 1.0 U/kg of BLM into *Dicer^fl/fl^* WT and *CD11c^Cre^Dicer^fl/fl^* KO mice. To provide a reference for non-injured lungs of *Dicer^fl/fl^* and *CD11c^Cre^Dicer^fl/fl^* groups, animals were given saline without BLM. Fibrosis was assessed 15 days later using mouse physical conditions, lung histology and hydroxyproline, and BAL TGF-β1 measurements. As expected, mouse body weights were gradually increased in both WT and DicerKO groups treated with saline (**Figure 4A**). However, the body weights of BLM-treated WT mice were significantly reduced with a peak of body weight loss (~20%) at day 10 after treatment followed by a gradual recovery afterwards, which is consistent with previous studies using BLM-treated mice (40, 41). Interestingly, there was markedly less weight loss observed in DicerKO mice following BLM treatment compared to BLM-treated WT mice. Histological analysis using H&E staining and trichrome staining of lungs from BLM-treated WT mice showed robust cell infiltration and heavy collagen deposition, typical of BLM-induced lung fibrosis (23) (**Figures 4B, C**). Similarly, smaller areas of damage with relatively milder inflammatory infiltrates and less collagen deposition were observed in *CD11c^Cre^Dicer^fl/fl^* KO mice compared to WT mice given BLM. Likewise, lungs from BLM-treated WT mice demonstrated a marked increase in hydroxyproline content and airway TGF-β1 levels compared to saline-treated WT mice, whereas lungs from BLM-treated DicerKO mice had dramatically reduced hydroxyproline and TGF-β1 compared to WT mice given BLM (**Figures 4D, E**). Taken together, these results suggest that deletion of miRNAs in AMs and other CD11c^+^ cells using CD11c-Cre-Dicer^fl/fl^ mice attenuates the development of BLM-induced fibrosis.

Using flow cytometry, we further analyzed lung immune profiles of both WT and *CD11c^Cre^Dicer^fl/fl^* KO mice following saline or BLM treatment (gating shown in **Supplementary Figures 2B, C**). In saline-treated mice, as expected, the number of TR-AMs was lower in DicerKO mice compared to WT mice and most of other immune cell types remained unaltered including MOs, DCs, neutrophils, eosinophils, B cells, interstitial macrophages (IMs) and, CD8^+^ T cells and regulatory T cells (Treg), (**Supplementary Figure 4**). Interestingly, we observed increased numbers of CD4^+^ T cells, natural killer T (NKT) cells and mucosal-associated invariant T (MAIT) cells in the lungs of DicerKO mice, suggesting that these cells may play a compensatory role during loss of TR-AMs at steady state. On the other hand, lungs isolated from BLM-treated DicerKO mice had significant decreased numbers of total cells and CD45^+^ immune cells compared to BLM-treated WT mice (**Supplementary Figure 4**), which is consistent with our observations from histological analysis (**Figure 4B**). These results suggest that deletion of miRNAs in AMs and other CD11c^+^ cells lessens both innate and adaptive immune responses induced by BLM injury. Further, our Ashcroft fibrosis scores showed a strong positive correlation with hydroxyproline levels, confirming consistency between histological and biochemical assessments of fibrosis in the BLM model. Furthermore, correlation analysis revealed a significant association between increasing Ashcroft scores and the TR-AM population in both BAL and lung, while no significant correlation was observed with Mo-AM populations, suggesting that macrophage heterogeneity (**Supplementary Figures 5A-C**), particularly TR-AM dynamics, is linked to the extent of bleomycin-induced lung injury.

### 2.5 Lack of miRNAs barely affects Mo-AM generation in lung fibrosis

In contrast to TR-AMs that can self-maintain in the steady state, Mo-AMs rapidly emerge in both airspace and lung tissue compartments as early as 4 days after BLM treatment and the number of Mo-AMs continues increasing over fibrotic progression (days 7–21 post-BLM) (28, 31). As shown in **Figures 4F, G**, we found a significant decrease in the number of TR-AMs (CD64^+^Siglec-F^hi^CD11b^lo^) in the BAL and lungs of *CD11c^Cre^Dicer^fl/fl^* KO mice compared to WT mice at day 15 post-BLM treatment. Meanwhile, we observed a comparable number of Mo-AMs (CD64^+^Siglec-F^lo^CD11b^hi^) in the airways and lungs of BLM-treated DicerKO mice compared to their WT counterparts. The expression levels of Dicer mRNA and macrophage-associated miRNAs were largely reduced in both TR-AMs and Mo-AMs isolated from BLM-treated *CD11c^Cre^Dicer^fl/fl^* KO mice (**Supplementary Figures 1A, B**), validating the efficacy of miRNA depletion in the two AM subgroups under fibrotic conditions. Additionally, as Ki-67 is a well-established marker of cell proliferation, as it is expressed in cells actively progressing through the cell cycle (G1, S, G2, and M phases). Hence, the use of Ki-67 is to delineate the proliferation of TR-AMs, as it reliably identifies proliferating cells. Both TR-AMs and Mo-AMs from BLM-treated WT and DicerKO mice had unaltered expression levels of Ki-67 protein (**Figures 4H, I**), while TR-AMs but not Mo-AMs exhibited increased frequency of annexin V^+^ apoptotic cells during fibrosis (**Figures 4J, K**). These results suggest that miRNAs are not only required for TR-AM steady-state homeostasis but are also essential for TR-AM maintenance during BLM-induced lung fibrosis, possibly through the regulation of TR-AM cell survival in both healthy and disease conditions. However, miRNA deficiency has negligible effect on the generation of Mo-AMs during lung fibrosis, suggesting that the fibrotic microenvironment can drive BM-derived cell differentiation into Mo-AMs regardless of miRNA status.

### 2.6 Transcriptomic landscape of TR-AMs and Mo-AMs with miRNA deficiency during BLM injury

**Figure 5:**
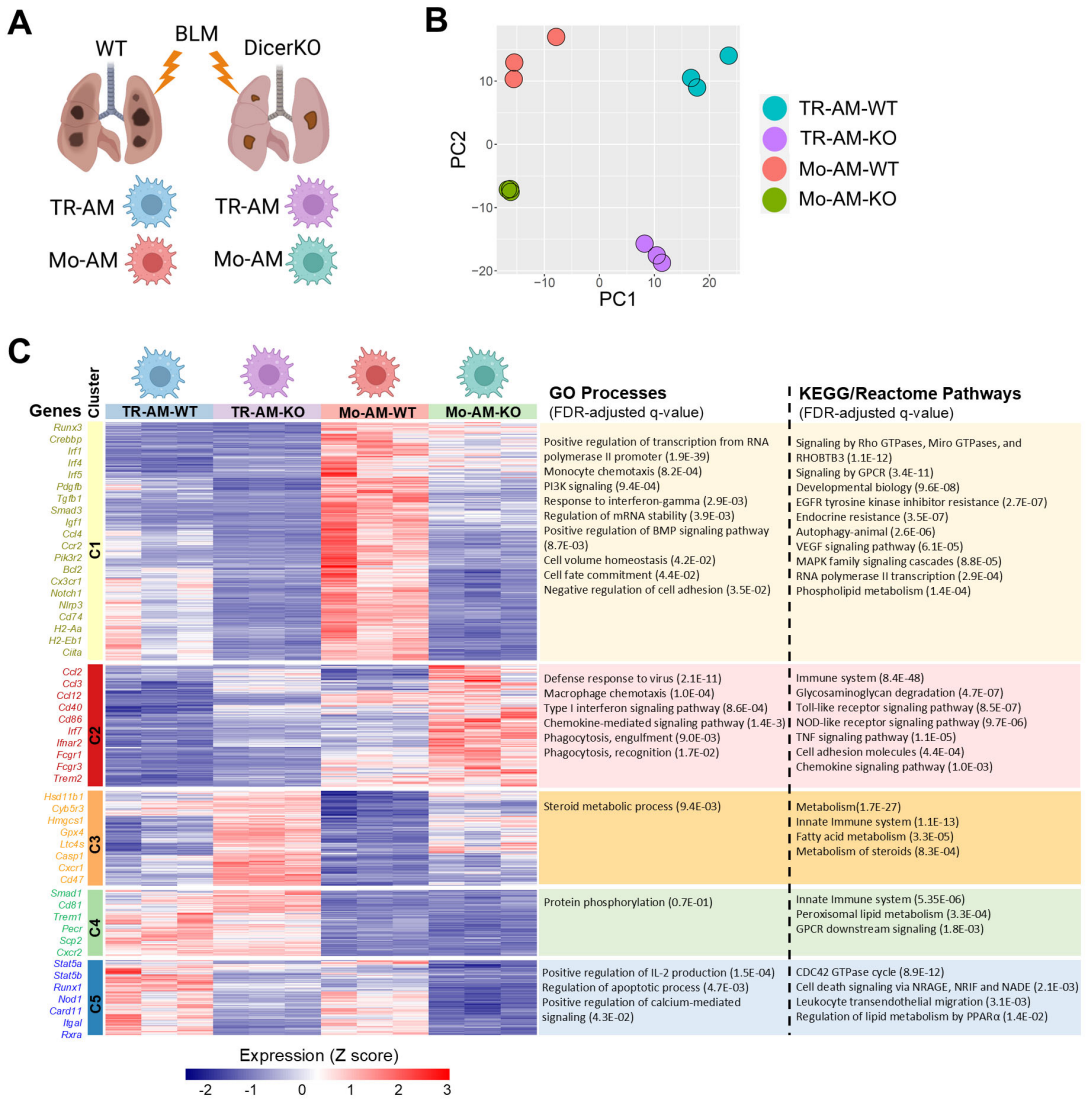
Lack of miRNAs impacts different pathways in TR-AMs and Mo-AMs after BLM injury. Transcriptional profiling of TR-AMs and Mo-AMs FACS-sorted from *Dicer^fl/fl^* (WT) and *Cd11c^cre^Dicer^fl/fl^* (KO) adult mice at day 14 after BLM treatment by bulk RNA-seq. **(A)** Experimental outline. **(B)** Principal-component analysis (PCA) of the transcriptomes of each AM subtype. **(C)** k-means clustering of all identified differentially expressed genes (FDR < 0.05 with Bonferroni correction) among 4 AM groups. The characteristic genes and gene ontology (GO) processes as well as KEGG/Reactome pathways associated with each cluster are shown on the left and right sides, correspondingly.

To better understand how miRNAs regulate the function of TR-AMs and Mo-AMs during lung fibrosis, we flow-sorted TR-AMs and Mo-AMs from *Dicer^fl/fl^* WT and *CD11c^Cre^Dicer^fl/fl^* KO mice two weeks after the administration of BLM and analyzed their gene expression using bulk RNA-seq (**Figure 5A**). Principle-component analysis of gene expression values showed clustering of the replicates according to cellular populations from two mouse strains given BLM (**Figure 5B**). A total of 2,689 differentially expressed genes (DEG) were identified among 4 AM groups (FDR < 0.05 with Bonferroni correction, **Supplementary Table 1**). We then performed k-means clustering of the entire transcriptional dataset of DEG, which revealed five distinct clusters of genes (**Figure 5C**, **Supplementary Table 2**). Cluster 1 contained core gene signatures that were highly enriched in WT Mo-AMs but were dramatically downregulated in DicerKO Mo-AMs. A significant portion of genes in this cluster were also moderately expressed in WT TR-AMs and barely expressed in DicerKO TR-AMs. Gene ontology and pathway analyses revealed genes in cluster 1 associated with transcription (*Runx3, Ahr, Crebbp, Irf1, Irf4*, and *Irf5*), cell adhesion (*Plxna1, Plxnb2, Plxnc1*, and *Plxnd1*), monocyte chemotaxis (*Ccl4, Ccl5, Ccl9*, and *Ccr2*), immune responses (*Nlrp3, Il1b, Cd74, H2-Aa, H2-Eb1*, and *Ciita*), autophagy (*Bcl2, Ulk1*, and *Atg2b*), as well as BMP (*Norch1* and *Norch2*), PI3K (*Pik3r2, Pik3cb, Pik3c2a, and Pik3cd*), GPCR (*Cx3cr1, Cxcr4*, and *Ccr5*), VEGF (*Akt3* and *Pik3r2*), and MAPK (*Il6, Csf2rb2*, and *Jak2*) signaling pathways. Several key genes implicated in fibrotic processes were also identified in this cluster, including *Pdgfb, Tgfb1, Smad3*, *Mmp12*, and *Igf1*. Cluster 2 contained a set of hub genes that were upregulated in DicerKO Mo-AMs while remaining almost unaltered in DicerKO TR-AMs compared to their WT control cells. Genes in cluster 2 were associated with immune defense (*Cd40, Cd86*, and *Irf7*), macrophage chemotaxis (*Ccl2, Ccl3*, and *Ccl12*), phagocytosis (*Fcgr1, Fcgr3*, and *Trem2*), as well as TLR (*Tlr1, Cd14*, and *Spp1*), NLR (*Jun, Nfkb1a*, and *Tank*), TNF (*Tnf, Fos*, and *Junb*) and type I IFN (*Ifnar2*, *Ifitm3*, and *Oas2*) signaling pathways. Cluster 3 included genes that were upregulated most significantly in DicerKO TR-AMs and moderately in DicerKO Mo-AMs compared to their WT counterparts. Genes in this cluster were involved in steroid metabolism (*Hsd11b1, Cyb5r3, Hmgcs1*, and *Sc5d*), fatty acid metabolism (*Gpx4, Ltc4s*, and *Cpt2*), and the innate immune system (*Casp1, Casp8, Cxcr1*, and *Cd47*). Cluster 4 contained genes highly enriched in TR-AMs but not Mo-AMs from both WT and DicerKO mice, and these genes were involved in protein phosphorylation (*Smad1, Camk2d*, and *Mak*), the innate immune system (*Cd81, Trem1*, and *Ear1*), peroxisomal lipid metabolism (*Pecr*, *Scp2*, and *Acox1*), and GPCR downstream signaling (*Cxcr2, Fpr1, Fpr2*, and *Ffar4*). Cluster 5 comprised genes downregulated in both TR-AMs and Mo-AMs from DicerKO mice compared to that from WT mice. Genes in this cluster were associated with regulation of IL-2 production (*Stat5a, Stat5b*, and *Runx1*), cell apoptosis/death (*Nod1, Card11, Net1*, and *Vav2*), migration (*Ctnnd1* and *Itgal*), and PPARα-regulated lipid metabolism (*Ncoa1, Ncor2*, and *Rxra*). Collectively, these data show that TR-AMs and Mo-AMs have distinct gene signatures in response to BLM injury and that loss of miRNAs impacts different pathways including apoptosis in the two AM subtypes during the development of fibrosis.

### 2.7 Dysregulated miRNA expression profiles in TR-AMs and Mo-AMs in BLM-induced lung fibrosis

**Figure 6:**
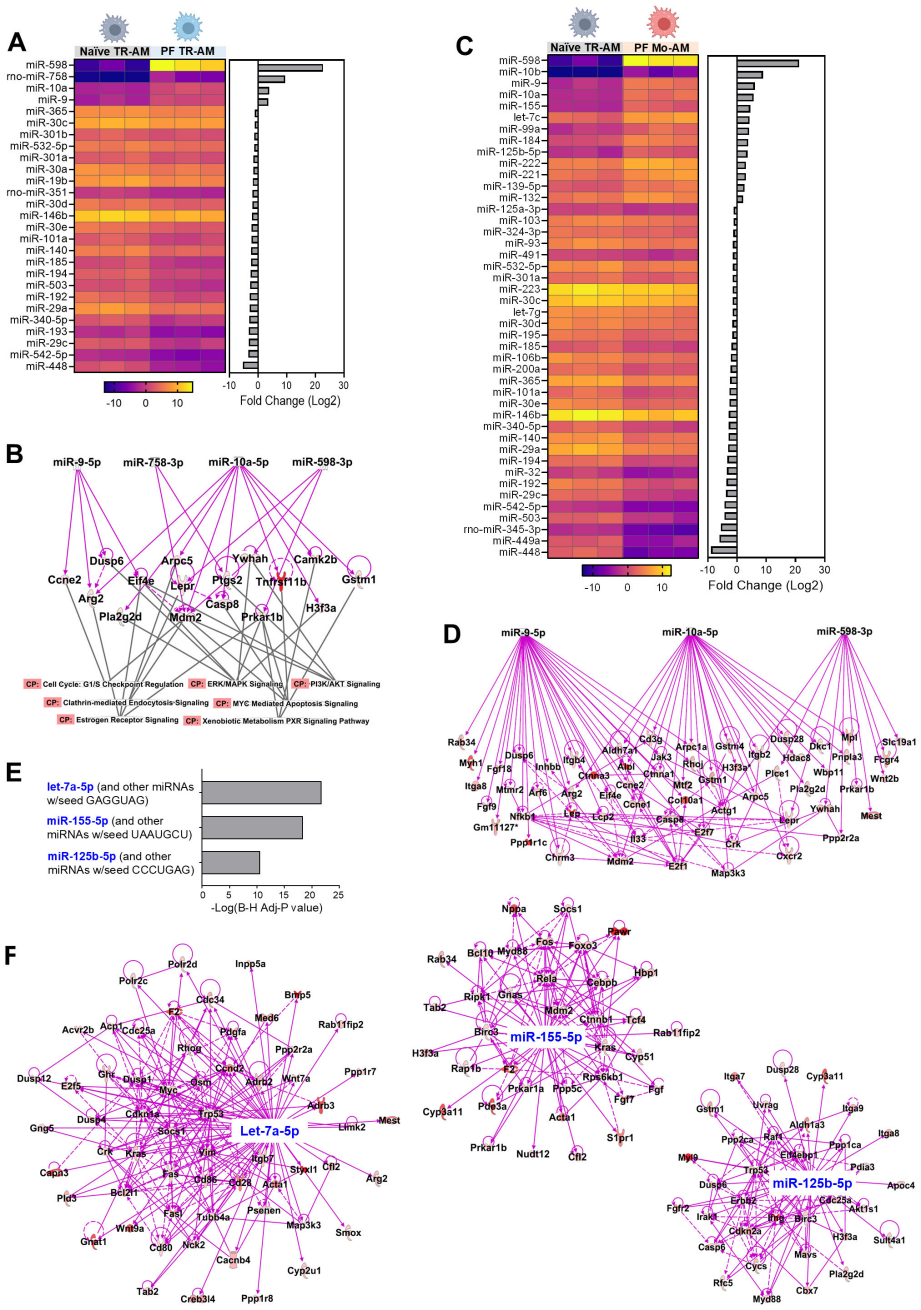
Putative miRNAs targeting genes that regulate TR-AMs and Mo-AMs after BLM injury. **(A)** Heatmap showing differential miRNA expression levels (-ddCt values) in naïve TR-AMs and responded TR-AMs from C57BL/6 mice given BLM. Bar chart showing fold changes (log2) of each miRNA by comparing responded TR-AMs to naïve TR-AMs on the right. **(B)** Ingenuity pathway analysis (IPA) analysis of differentially expressed genes **(DEG)** in DicerKO TR-AMs and associated signaling pathways targeted by 4 upregulated miRNAs as in **(A)** CP, canonical pathways. **(C)** Heatmap showing differential miRNA expression levels (-ddCt values) in naïve TR-AMs and responded Mo-AMs from C57BL/6 mice given BLM. Bar chart showing fold changes (log2) of each miRNA by comparing responded Mo-AMs to naïve TR-AMs on the right. **(D)** IPA analysis of the of DEG in DicerKO Mo-AMs targeted by 3 upregulated miRNAs that were shared in **A** and **(C, E)** Predicated upstream miRNA regulators in Mo-AMs by IPA. **(F)** IPA analysis of DEG targeted by predicated upstream regulator miRNAs.

Hundreds of miRNAs have been identified in mammalian AMs in both steady-state and pathological conditions (42, 43). To investigate which individual miRNAs are involved in AM regulation during lung fibrosis, we analyzed miRNA expression profiles of TR-AMs purified from naïve WT mice as well as responded TR-AMs and Mo-AMs purified from BLM-treated WT mice using miRNA array. A total of 27 miRNAs were differentially expressed in the responded TR-AMs compared to naïve TR-AMs, including 4 upregulated miRNAs and 23 downregulated miRNAs (**Figure 6A**). It is known that miRNAs can negatively regulate gene expression by suppressing gene translation and promoting mRNA degradation (44). Most of the changes induced by global miRNA loss (DicerKO system) occur at the level of transcription (45). We then integrated miRNA expression patterns from miRNA array with gene expression profiles identified by bulk RNA-seq and particularly focused on the upregulated miRNAs in AMs from fibrotic tissues which may shut down the gene expression during fibrosis, and the upregulated DEG in DicerKO AM populations from fibrotic lungs, which could be impacted by the lack of miRNAs. The 4 miRNAs that were significantly upregulated in TR-AMs following BLM injury include miR-598, miR-758, miR-10a, and miR-9. Ingenuity pathway analysis (IPA) revealed that these 4 miRNAs potentially regulated a group of DEG identified in miRNA-deficient TR-AMs during BLM injury that were related to canonical pathways involved in cell cycle, cell death, metabolism, and immune responses (**Figure 6B**, **Supplementary Table 3**). Likewise, a total of 44 miRNAs were differentially expressed in the responded Mo-AMs compared to naïve TR-AMs, including 13 upregulated miRNAs and 31 downregulated miRNAs (**Figure 6C**). We found that miR-598, miR-10a, and miR-9 were also upregulated in the responded Mo-AMs and potentially impacted a substantial number of DEG in the miRNA-deficient Mo-AMs during lung fibrosis (**Figure 6D**, **Supplementary Table 4**). Interestingly, IPA predicted several upstream regulator miRNAs, including let-7a (sharing the same seeding sequence, GAGGUAG, with let-7c), miR-155, and miR-125b (**Figure 6E**), which were also specifically upregulated in the responded Mo-AMs (**Figure 6C**). These putative upstream miRNAs possibly directly or indirectly regulate DEG associated with dysregulated pathways in DicerKO Mo-AMs during BLM injury (**Figure 6F**, **Supplementary Table 5**). Taken together, these results suggest that BLM injury caused similar but divergent miRNA expression changes in TR-AMs and Mo-AMs, and that several miRNAs specifically regulated in Mo-AMs during fibrosis might be responsible for Mo-AM-specific functional changes in response to BLM injury.

## 3 Discussion

As the resident macrophages in the airways, AMs are essential for maintaining lung tissue homeostasis through clearance of surfactant and cell debris under physiological conditions as well as regulating host defense against infection, stress, and injury under pathological conditions (46). miRNAs are a cluster of small non-coding RNAs that are important epigenetic regulators of immune cell development, maintenance, and function. Our recent study demonstrates that miRNAs are required for embryonic AM development (19); however, it remains unclear whether misregulation of miRNAs can influence AM homeostasis and function in pulmonary diseases. Here, we show that miRNAs are required for TR-AM postnatal maintenance and maturation in the steady state. miRNAs also control AM repopulation from BM progenitors after lethal whole-body irradiation. In a BLM-induced experimental PF model, miRNA deficiency leads to distinct transcriptomic and pathway changes in TR-AMs and Mo-AMs in response to fibrotic injury, which could possibly contribute to the attenuated development of fibrogenesis. Our results suggest miRNAs as a critical regulator for AM behaviors in health and disease.

In addition to TR-AMs, the most abundant macrophage population in the lungs, the second macrophage subset IMs reside in the lung interstitium and can be distinguished from TR-AMs according to their anatomical location, morphology, and phenotype (47). However, the origin of IMs and their function in steady and pathogenic states remain less studied. Phenotypically, TR-AMs are autofluorescent Siglec F^hi^CD11c^hi^CD11b^lo^CX3CR1^–^ cells, while IMs are non-autofluorescent SiglecF^–^CD11c^–/lo^CD11b^hi^CX3CR1^+^ cells. Due to little expression of CD11c by IMs, gene deletion induced by CD11c-Cre barely targets IMs (38). Interestingly, we observed an increase in the number of IMs in *CD11c^Cre^Dicer^fl/fl^* KO naïve mice compared to *Dicer^fl/fl^* control naïve mice, suggesting that IMs may compensatorily increase due to the loss of TR-AMs in the DicerKO mice in the steady state. On the other hand, the CD11c-Cre promoter also targets DCs which highly express CD11c (48). DCs have been implicated to play a role in BLM-induced lung injury since inactivation of DCs by immune-mediator VAG539 and depletion of DCs by CD11c-diphtheria toxin receptor mitigates lung fibrosis, although the targets of both approaches are not restricted to DCs (49, 50). Even though we did not see changes in the numbers of DCs in the lungs of naïve DicerKO mice generated using the CD11c promoter, we observed reduced numbers of DCs in the fibrotic lungs of DicerKO mice given BLM. Thus, we cannot exclude the possibility that some of the lessened effects we observed are attributable to DCs. Additionally, lack of miRNAs led to decreased numbers of TR-AMs in the BAL and lungs from both naïve and BLM-treated mice. As TR-AMs are an important source of fibroblast growth factor that drive fibrogenesis during PF (51), the lower quantity of TR-AMs may also contribute to the reduced PF severity in the DicerKO mice.

TR-AMs can self-renew in adult mice independent of BM input in the steady state. However, under certain stresses that induce significant TR-AM loss, the lungs are replenished with fresh Mo-AMs from BM-derived MOs (52). BM chimeras have proven to be a valuable tool to study hematopoietic cell development and their participation in long-term immune responses in a physiological setting (53). In our study, BM cells from DicerKO mice failed to generate Mo-AMs in the BM chimeric mice following lethal irradiation, indicating that miRNAs are required for Mo-AM repopulation from BM under physiological conditions. Interestingly, during BLM-induced lung injury, comparable numbers of Mo-AMs emerged in the airways and lungs between DicerKO and WT mice, implying that loss of miRNAs barely influences the generation of Mo-AMs under inflammation. One possibility for this discrepancy is that the local environmental cues elicited by BLM injury may drive MOs to differentiate into Mo-AMs in the inflamed tissues. The cytokine GM-CSF is essential for the differentiation of fetal MOs into TR-AMs through induction of the expression of PPAR-γ, a key transcription factor required for TR-AM embryonic development and Mo-AM replenishment after irradiation-induced depletion (7). A previous study found a significant increase in the GM-CSF mRNA level in the lungs on day 5 after BLM instillation, and that the percentage and number of AMs within BAL fluid increased following GM-CSF infusion and decreased following anti-GM-CSF IgG treatment (54). These results suggest that GM-CSF induced by BLM injury may promote Mo-AM differentiation in inflamed lungs. Moreover, our bulk RNA-seq data showed a mild increase (1.34-folds change, FDR = 0.049) in the PPAR-γ mRNA level and comparable mRNA levels of GM-CSF receptors (*Csf2ra* and *Csf2rb*) of Mo-AMs from Dicer cKO mice compared to the WT control, suggesting that BLM-induced GM-CSF could efficiently drive the differentiation of MOs into Mo-AMs in the absence of miRNA regulation. Future studies using anti-GM-CSF antibody treatment of DicerKO mice given BLM may address whether there is a dependency of GM-CSF for Mo-AM generation in the lung fibrosis model. On the other hand, deletion of Dicer leads to a significant loss of almost all miRNAs in the Dicer-deficient cells (55). Although the generation of Mo-AMs appears to not be affected by Dicer deletion during BLM-PF, we cannot rule out the possibility that some individual miRNAs may still play a role in Mo-AM production under fibrosis disease conditions, and that deficiency of these miRNAs in DicerKO mice may offset their promoting or inhibitory functions in the generation of Mo-AMs from BM during fibrogenesis. This concern could be addressed using individual miRNA conditional KO or overexpressing (knock-in) mouse strains with the experimental PF model.

BLM-induced lung injury is a self-limiting process characterized by a profound initial inflammatory phase (0–7 days), a progressive fibrogenic phase (7–21 days), and a spontaneous resolution phase (42–56 days) (34, 56). Depletion of TR-AMs by clodronate liposomes either accelerates or attenuates acute pulmonary damage induced by a variety of toxicants or mechanical disruption, suggesting that the pro/anti-inflammatory role of TR-AMs in the acute inflamed lungs is context dependent (57–59). Moreover, the role of TR-AMs in the fibrotic phase of BLM injury remains debatable (28, 29, 60), although the profibrotic role of Mo-AMs in developing BLM-induced fibrosis has been firmly established (29–31). There is also evidence indicating that depletion of lung macrophages during the resolution phase of BLM-PF slows the resolution of fibrosis (33, 34). Thus, the roles of TR-AMs and Mo-AMs could be distinct in the different phases of lung injury and repair. In the current study, we showed that loss of miRNAs in AMs and other CD11c^+^ cells attenuated lung fibrogenesis at day 14–15 after BLM treatment. However, whether miRNAs regulate AM function at the initial inflammatory and resolution phases remains unclear. Inducible gene deletion models, such as CD11cCreER_T_ mice (61), could be used to induce timed deletion of miRNAs in AMs and evaluate the role of miRNAs in AM function at different phases of BLM-induced lung injury. One may also argue that CD11c promoter-driven Cre activity occurs in both TR-AMs and Mo-AMs, which vaguely defines the specific role of TR-AMs and Mo-AMs with miRNA deficiency contributing to the lessened fibrogenesis. This concern could be addressed by using recently developed transgenic mice that specifically target TR-AMs (e.g., CD169-Cre) (62–64) or Mo-AMs (e.g., hCD68rtTA-tet-On-Cre) (31, 65) in the experimental lung disease models. Despite the limitation using CD11c-Cre mice, our bulk RNA-seq analysis demonstrated divergent transcriptomic changes and differentially impacted pathways in TR-AMs and Mo-AMs purified from the fibrotic lungs of miRNA-deficient mice, which provides some interesting clues on miRNA differential regulation in AM subtype behaviors during lung fibrosis.

Using an integration of miRNA array, mRNA-seq, and bioinformatics analyses, we identified several miRNAs and their potential targeted mRNAs and pathways that possibly contribute to AM-mediated fibrogenesis during BLM injury. Among potential pathway, the increased apoptosis observed in DicerKO TR-AMs can be linked to the transcriptomic alterations identified in our RNA-seq analysis. Notably, Cluster 5 contained several apoptosis- and survival-related genes (*Nod1*, *Card11*, *Net1*, *Vav2*) that were downregulated in both TR-AMs and Mo-AMs from DicerKO mice, suggesting a loss of pro-survival signaling pathways. In addition, Cluster 1 featured key transcriptional and fibrotic regulators (*Runx3*, *Smad3*, *Tgfb1*) that were diminished in Dicer-deficient TR-AMs, potentially impairing homeostatic and repair functions. Cluster 3 included genes involved in lipid and steroid metabolism (*Hsd11b1*, *Cpt2*, *Gpx4*) and innate immune activation (*Casp1*, *Cd47*), indicating a shift in metabolic and immune tone that may further sensitize TR-AMs to apoptotic stress. Conversely, genes in Cluster 4, which were selectively enriched in TR-AMs, may represent a core identity signature that is maintained, but insufficient to compensate for the apoptotic pressures triggered by miRNA loss. Together, these data suggest that Dicer deletion disrupts a network of survival, metabolic, and immune-regulatory pathways that collectively contribute to the heightened apoptosis observed in TR-AMs during fibrotic injury. An important aspect of TR-AM depletion in Dicer KO mice is the potential role of repopulating Mo-AMs, which may influence TR-AM depletion due to their distinct origin and roles in lung injury. Mo-AMs, recruited from the bone marrow during inflammation, play a key role in immune responses, while TR-AMs are tissue-resident macrophages that maintain lung homeostasis and respond to local injury. This distinction may contribute to differential effects on macrophage populations during lung fibrosis. While our study primarily focused on miRNA loss in TR-AMs and their association with increased apoptosis, Mo-AMs, recruited to the lungs during injury and inflammation, could contribute to the depletion of TR-AMs. These Mo-AMs may secrete pro-apoptotic factors or other molecules that exacerbate TR-AM loss, potentially through a paracrine mechanism. The interplay between TR-AMs and Mo-AMs in the context of Dicer loss and fibrosis is an exciting area for future research. Furthermore, miR-598, miR-10a, and miR-9 were significantly upregulated in both TR-AMs and Mo-AMs following BLM and potentially targeted a group of DEG shared by TR-AMs and Mo-AMs, such as *Arg2, Casp8, Ccne2, Dusp6, Eif4e, Gstm1*, *H3f3a, Lepr, Mdm2, Prkar1b*, and *Ywhah*. These shared DEG are involved in multiple signaling pathways that are associated with the pathogenesis of lung fibrosis, including G1/S checkpoint regulation, Myc-mediated apoptosis, clathrin-mediated endocytosis, ERK/MAPK, and PI3K/AKT signaling pathways. On the other hand, let-7c (sharing the same seeding sequence with let-7a), miR-125b, and miR-155 uniquely upregulated in Mo-AMs during BLM injury were predicted as putative upstream regulators governing transcriptomic and pathway changes in DicerKO Mo-AMs. The levels of let-7c, miR-125b, and miR-155 were significantly increased in the lung biopsies from rapidly progressive IPF patients compared to normal lung samples (66), suggesting that their aberrant regulation might correlate to severity of IPF disease progression. Furthermore, let-7c levels were reported greater in AMs from fibrotic lungs after BLM treatment as compared to those from normal lungs (67), which is consistent with our results. Although extensive gain- or loss-of-function studies have revealed that let-7c and miR-125b have major impact on BM-derived macrophage polarization and activation (67–70), whether they are essential for regulating Mo-AM profibrotic phenotypes during experimental PF remains unclear. On the other hand, miR-155 has been reported to promote pro-inflammatory activation of BM-derived macrophages and inhibit the polarization of anti-inflammatory and repair macrophage phenotypes (71). However, the role of miR-155 in the regulation of AMs in BLM-induced lung fibrosis remains debated. Kurowska-Stolarska, M. et al. reported that miR-155^–/–^ conventional KO mice given a single dose of BLM developed exacerbated lung fibrosis, increased collagen deposition, TGF-β production, and activation of profibrotic macrophages by dysregulation of liver X receptor (LXR)α in lung fibroblasts and macrophages (72). Christmann, R.B. et al. reported that miR-155KO mice chronically exposed to BLM developed milder chronic lung fibrosis, better survival rates, and lower expression of BLM-induced lung profibrotic genes, such as Arginase-1 and tissue inhibitor of metalloproteinases-1, both of which were produced prominently by lung macrophages (73, 74). Thus, miR-155 appears to play either pro- or anti-fibrotic role in the pathogenesis of lung fibrosis. This discrepancy may result from different experimental setups of BLM treatment in the two studies, i.e., 2.4 U/kg for 1 dose by intranasal injection versus 90 U/kg for 7 days by subcutaneous osmotic pump injections. Additionally, global deletion of miR-155 could compromise its role in AMs during BLM injury given the overall effects of miR-155 deficiency on fibroblasts and other immune cell types, such as IMs, DCs and T cells. This notion could be addressed by future functional experiments using transgenic mice with AM-specific deletion or overexpression of individual miRNAs. Considering the time-consuming process of generating new mouse strains, we believe that *in vivo* local delivery of miRNA mimics or antagomirs via liposomes or nanoparticles specifically targeting AMs would provide a promising and more efficient strategy for AM functional studies in lung disease models. Specifically, we plan to perform targeted inhibition of selected miRNAs followed by apoptosis assays in TR-AMs to further elucidate their functional relevance (60, 75).

In conclusion, our data have provided evidence that miRNAs serve as key epigenetic regulator to control AM maintenance, maturation, and repopulation from BM under physiological conditions. More importantly, our finding that miRNAs are involved in the regulation of AM survival and function during the development of lung fibrosis may provide additional knowledge about miRNA regulation in AM biology and may suggest modulation of AMs using miRNAs for clinical applications to treat IPF.

## 4 Methods

### 4.1 Animals

C57BL/6 (strain #000664), B6.SJL (strain #002014), *Cd11c^Cre^* (strain #008068), and *Dicer^fl/fl^* (strain #006001) mice were purchased from the Jackson Laboratory (Bar Harbor, ME). To generate CD11c-expressing, cell-specific Dicer mutant mice (*Cd11c^Cre^Dicer^flfl^*), we crossed *Cd11c^Cre^* and *Dicer^fl/fl^* mice. All experiments included age-matched and sex-matched littermate controls of 6–18 weeks of age. Mice were housed under barrier- and specific pathogen-free conditions at temperatures of 20-26 °C with 30-70% humidity and a 12h light/12h dark cycle at Henry Ford Health System (HFHS). All procedures were approved by the HFHS Institutional Animal Care and Use Committee (Protocol No.1478) and conformed to the Guide for the Care and Use of Laboratory Animals published by the U.S. National Institutes of Health.

### 4.2 Bleomycin injury

Bleomycin (Sigma, catalog #B8416) was instilled intratracheally at a dose of 0.5-3.0 U/kg in 40 µL of saline using an established protocol (76). Briefly, anesthetized mice (45 mg/kg ketamine and 8 mg/kg xylazine) were suspended on a 60-degree incline board. With the tongue gently extended, a 40 ul aliquot of bleomycin in saline or saline alone was placed in the back of the oral cavity to be aspirated by the animal. To guide the solution down the trachea rather than the esophagus, nasal breathing was blocked by covering the nose until the full volume was consumed. The treated mice were then placed in the original cage and kept monitoring until full recovery from anesthesia.

### 4.3 Assessing fibrosis

To assess lung fibrosis development and severity, mouse body weight loss and survival were monitored over a 2-week period. Additionally, lung tissue was harvested for hydroxyproline analysis and H&E and trichome staining. Tissue samples were homogenized or fixed in 10% formalin, respectively. Lung hydroxyproline content was quantified using the colorimetric Hydroxyproline Assay Kit (Abcam, catalog # Ab222941), while H&E- and trichome-stained slides were imaged using the Olympus FSX100 Inverted Microscope (Olympus) and FSX-BSW image capturing software v.03.02.12 (Olympus). TGF-β1 levels in BAL were measured using Human/mouse TGF beta 1 uncoated ELISA Kit (Invitrogen, catalog# 88-8350). Further, Hematoxilin Eosin-stained slides were scanned with light microscope (Leica, Germany) at 100× magnification for images, reviewed and degree of fibrosis was assigned according to Ashcroft’s fibrosis score system (77): normal lung was referred to as score “0” while score “8” represented maximal degree. Later, degree of Ashcroft’s fibrosis score was correlated with hydroxyproline, TR-AM and Mo-AM to determine their association with fibrotic progression.

### 4.4 Bulk RNA sequencing and data processing

cDNA synthesis and preamplification for total RNA of the sorted cells were performed using the SMART-Seq v4 Ultra Low Input RNA Kit (Clontech, Mountain View, CA) following the manufacturer’s instructions as described previously (13). Illumina P5 and P7 primers with adaptors were used for final library amplification. Sequencing was performed using paired-end 150 bp reads on the Illumina Hiseq 4000 platform from Novagene. The RNA-Seq reads were aligned to the mouse GRCm38/mm10 reference genome and expression levels for each sample were quantified and normalized using Biomedical Genomics Workbench 5.0 (QIAGEN). Differentially expressed gene analysis was performed in R (4.1.2) using the DESeq2 package (1.37.0). Principle-component analysis was performed in R (4.1.2) using the ggplot2 package (3.3.5). Heatmap was created in R (4.1.2) using the pheatmap package (1.0.12). Gene ontology (GO) biological process enrichment analysis as well as KEGG and Reactome pathway analyses were performed using DAVID Bioinformatics Resources 6.8 (78). Integration of miRNA and DEG interaction analysis was performed using the Ingenuity Pathway Analysis (QIAGEN Bioinformatics, version 42012434).

### 4.5 Bone marrow chimeras

B6.SJL (CD45.1^+^) mice were lethally irradiated with 550 Rads for 2 times. Donor BM cells were harvested from age- and sex-matched CD45.2^+^ *Dicer^fl/fl^* (WT) and *Csf1r^icre^Dicer^fl/fl^* (KO) mice by flushing bones followed by lysis of erythrocytes. The BM cells (10^7^ cells in 100 μl PBS) from either *Dicer^fl/f l^* (WT) or *Csf1r^icre^Dicer^fl/fl^* (KO) mice was retro-orbitally administered into each irradiated recipient mouse. The recipient mice were kept for 10 weeks to allow for reconstitution, after which they were used for experiments.

### 4.6 Statistics

No statistical method was utilized to predetermine sample size. Student’s unpaired two-tailed *t* tests were performed using GraphPad Prism 8.4.3 software (GraphPad, La Jolla, CA) unless otherwise specified. Statistical significance is displayed as: *N.S.*, not significant; *, *P* < 0.05; **, *P* < 0.01; ***, *P* < 0.001.

### 4.7 Study approval

All animal procedures were approved and performed in accordance with the ethics guidelines of Henry Ford Health System Animal Care and Use Committee.

## Data Availability

The data presented in the study have been deposited in the Gene Expression Omnibus (GEO) under accession number GSE199886.

## References

[1] Hussell T Bell TJ. Alveolar macrophages: plasticity in a tissue-specific context. Nat Rev Immunol. (2014) 14:81–93. doi: 10.1038/nri3600 24445666

[2] Schulz C Gomez Perdiguero E Chorro L Szabo-Rogers H Cagnard N Kierdorf K. A lineage of myeloid cells independent of myb and hematopoietic stem cells. Science. (2012) 336:86–90. doi: 10.1126/science.1219179 22442384

[3] Perdiguero EG Klapproth K Schulz C Busch K de Bruijn M Rodewald HR. The origin of tissue-resident macrophages: when an erythro-myeloid progenitor is an erythro-myeloid progenitor. Immunity. (2015) 43:1023–4. doi: 10.1016/j.immuni.2015.11.022 26682973

[4] Hoeffel G Chen J Lavin Y Low D Almeida FF See P. C-myb(+) erythro-myeloid progenitor-derived fetal monocytes give rise to adult tissue-resident macrophages. Immunity. (2015) 42:665–78. doi: 10.1016/j.immuni.2015.03.011 PMC4545768 25902481

[5] Mass E Ballesteros I Farlik M Halbritter F Gunther P Crozet L. Specification of tissue-resident macrophages during organogenesis. Science. (2016) 353:1–32. doi: 10.1126/science.aaf4238 PMC5066309 27492475

[6] Kopf M Schneider C Nobs SP. The development and function of lung-resident macrophages and dendritic cells. Nat Immunol. (2015) 16:36–44. doi: 10.1038/ni.3052 25521683

[7] Schneider C Nobs SP Kurrer M Rehrauer H Thiele C Kopf M. Induction of the nuclear receptor ppar-gamma by the cytokine gm-csf is critical for the differentiation of fetal monocytes into alveolar macrophages. Nat Immunol. (2014) 15:1026–37. doi: 10.1038/ni.3005 25263125

[8] Guilliams M De Kleer I Henri S Post S Vanhoutte L De Prijck S. Alveolar macrophages develop from fetal monocytes that differentiate into long-lived cells in the first week of life via gm-csf. J Exp Med. (2013) 210:1977–92. doi: 10.1084/jem.20131199 PMC3782041 24043763

[9] Hashimoto D Chow A Noizat C Teo P Beasley MB Leboeuf M. Tissue-resident macrophages self-maintain locally throughout adult life with minimal contribution from circulating monocytes. Immunity. (2013) 38:792–804. doi: 10.1016/j.immuni.2013.04.004 23601688 PMC3853406

[10] Amit I Winter DR Jung S. The role of the local environment and epigenetics in shaping macrophage identity and their effect on tissue homeostasis. Nat Immunol. (2016) 17:18–25. doi: 10.1038/ni.3325 26681458

[11] Roth P Dominguez MG Stanley ER. The effects of colony-stimulating factor-1 on the distribution of mononuclear phagocytes in the developing osteopetrotic mouse. Blood. (1998) 91:3773–83. doi: 10.1182/blood.V91.10.3773 9573014

[12] Yu X Buttgereit A Lelios I Utz SG Cansever D Becher B. The cytokine tgf-beta promotes the development and homeostasis of alveolar macrophages. Immunity. (2017) 47:903–12 e4. doi: 10.1016/j.immuni.2017.10.007 29126797

[13] Yao Y Liu Q Adrianto I Wu X Glassbrook J Khalasawi N. Histone deacetylase 3 controls lung alveolar macrophage development and homeostasis. Nat Commun. (2020) 11:3822. doi: 10.1038/s41467-020-17630-6 32732898 PMC7393351

[14] Tomankova T Petrek M Kriegova E. Involvement of micrornas in physiological and pathological processes in the lung. Respir Res. (2010) 11:159. doi: 10.1186/1465-9921-11-159 21092244 PMC3001429

[15] Sayed D Abdellatif M. Micrornas in development and disease. Physiol Rev. (2011) 91:827–87. doi: 10.1152/physrev.00006.2010 21742789

[16] Bernstein E Kim SY Carmell MA Murchison EP Alcorn H Li MZ. Dicer is essential for mouse development. Nat Genet. (2003) 35:215–7. doi: 10.1038/ng1253 14528307

[17] Seo KH Zhou L Meng D Xu J Dong Z Mi QS. Loss of micrornas in thymus perturbs invariant nkt cell development and function. Cell Mol Immunol. (2010) 7:447–53. doi: 10.1038/cmi.2010.49 PMC4002964 20852654

[18] Zhou L Park JJ Zheng Q Dong Z Mi Q. Micrornas are key regulators controlling inkt and regulatory T-cell development and function. Cell Mol Immunol. (2011) 8:380–7. doi: 10.1038/cmi.2011.27 PMC4012887 21822298

[19] Zhou L Seo KH He HZ Pacholczyk R Meng DM Li CG. Tie2cre-induced inactivation of the mirna-processing enzyme dicer disrupts invariant nkt cell development. Proc Natl Acad Sci United States America. (2009) 106:10266–71. doi: 10.1073/pnas.0811119106 PMC2700920 19509335

[20] Zhou L Seo KH Wong HK Mi QS. Micrornas and immune regulatory T cells. Int Immunopharmacol. (2009) 9:524–7. doi: 10.1016/j.intimp.2009.01.017 19539573

[21] Yao Y Martin C Yin C Guo C Dong Z Zhou L. Mirnas are required for langerhans cell, skin and lung resident macrophage ontogeny. J Allergy Clin Immunol. (2018) 143(2):976–8.e2. doi: 10.1016/j.jaci.2018.04.024 PMC6129406 29751006

[22] Fernandez IE Eickelberg O. New cellular and molecular mechanisms of lung injury and fibrosis in idiopathic pulmonary fibrosis. Lancet. (2012) 380:680–8. doi: 10.1016/S0140-6736(12)61144-1 22901889

[23] Moore BB Hogaboam CM. Murine models of pulmonary fibrosis. Am J Physiol Lung Cell Mol Physiol. (2008) 294:L152–60. doi: 10.1152/ajplung.00313.2007 17993587

[24] Pandit KV Milosevic J Kaminski N. Micrornas in idiopathic pulmonary fibrosis. Transl Res. (2011) 157:191–9. doi: 10.1016/j.trsl.2011.01.012 21420029

[25] Rajasekaran S Rajaguru P Sudhakar Gandhi PS. Micrornas as potential targets for progressive pulmonary fibrosis. Front Pharmacol. (2015) 6:254. doi: 10.3389/fphar.2015.00254 26594173 PMC4633493

[26] Raver-Shapira N Marciano E Meiri E Spector Y Rosenfeld N Moskovits N. Transcriptional activation of mir-34a contributes to P53-mediated apoptosis. Mol Cell. (2007) 26:731–43. doi: 10.1016/j.molcel.2007.05.017 17540598

[27] Byrne AJ Maher TM Lloyd CM. Pulmonary macrophages: A new therapeutic pathway in fibrosing lung disease? Trends Mol Med. (2016) 22:303–16. doi: 10.1016/j.molmed.2016.02.004 26979628

[28] Misharin AV Morales-Nebreda L Reyfman PA Cuda CM Walter JM McQuattie-Pimentel AC. Monocyte-derived alveolar macrophages drive lung fibrosis and persist in the lung over the life span. J Exp Med. (2017) 214:2387–404. doi: 10.1084/jem.20162152 PMC5551573 28694385

[29] Li D Guabiraba R Besnard AG Komai-Koma M Jabir MS Zhang L. Il-33 promotes st2-dependent lung fibrosis by the induction of alternatively activated macrophages and innate lymphoid cells in mice. J Allergy Clin Immunol. (2014) 134:1422–32 e11. doi: 10.1016/j.jaci.2014.05.011 24985397 PMC4258609

[30] Ji WJ Ma YQ Zhou X Zhang YD Lu RY Sun HY. Temporal and spatial characterization of mononuclear phagocytes in circulating, lung alveolar and interstitial compartments in a mouse model of bleomycin-induced pulmonary injury. J Immunol Methods. (2014) 403:7–16. doi: 10.1016/j.jim.2013.11.012 24280595

[31] McCubbrey AL Barthel L Mohning MP Redente EF Mould KJ Thomas SM. Deletion of C-flip from cd11b(Hi) macrophages prevents development of bleomycin-induced lung fibrosis. Am J Respir Cell Mol Biol. (2018) 58:66–78. doi: 10.1165/rcmb.2017-0154OC 28850249 PMC5941310

[32] Perdiguero EG Geissmann F. The development and maintenance of resident macrophages. Nat Immunol. (2016) 17:2–8. doi: 10.1038/ni.3341 26681456 PMC4950995

[33] Cabrera S Maciel M Hernandez-Barrientos D Calyeca J Gaxiola M Selman M. Delayed resolution of bleomycin-induced pulmonary fibrosis in absence of mmp13 (Collagenase 3). Am J Physiol Lung Cell Mol Physiol. (2019) 316:L961–L76. doi: 10.1152/ajplung.00455.2017 30785343

[34] Gibbons MA MacKinnon AC Ramachandran P Dhaliwal K Duffin R Phythian-Adams AT. Ly6chi monocytes direct alternatively activated profibrotic macrophage regulation of lung fibrosis. Am J Respir Crit Care Med. (2011) 184:569–81. doi: 10.1164/rccm.201010-1719OC 21680953

[35] Tashiro J Rubio GA Limper AH Williams K Elliot SJ Ninou I. Exploring animal models that resemble idiopathic pulmonary fibrosis. Front Med (Lausanne). (2017) 4:118. doi: 10.3389/fmed.2017.00118 28804709 PMC5532376

[36] Ono M Miyamura M Kyotani S Saibara T Ohnishi S Nishioka Y. Effects of sho-saiko-to extract on liver fibrosis in relation to the changes in hydroxyproline and retinoid levels of the liver in rats. J Pharm Pharmacol. (1999) 51:1079–84. doi: 10.1211/0022357991773429 10528993

[37] Kliment CR Englert JM Crum LP Oury TD. A novel method for accurate collagen and biochemical assessment of pulmonary tissue utilizing one animal. Int J Clin Exp Pathol. (2011) 4:349–55. PMC3093059 21577320

[38] Branchett WJ Cook J Oliver RA Bruno N Walker SA Stolting H. Airway macrophage-intrinsic tgf-beta1 regulates pulmonary immunity during early-life allergen exposure. J Allergy Clin Immunol. (2021) 147:1892–906. doi: 10.1016/j.jaci.2021.01.026 PMC8098862 33571538

[39] Eddy WE Gong KQ Bell B Parks WC Ziegler SF Manicone AM. Stat5 is required for cd103(+) dendritic cell and alveolar macrophage development and protection from lung injury. J Immunol. (2017) 198:4813–22. doi: 10.4049/jimmunol.1601777 PMC5624516 28500076

[40] Russo RC Garcia CC Barcelos LS Rachid MA Guabiraba R Roffe E. Phosphoinositide 3-kinase gamma plays a critical role in bleomycin-induced pulmonary inflammation and fibrosis in mice. J Leukoc Biol. (2011) 89:269–82. doi: 10.1189/jlb.0610346 21048214

[41] Chang J Nie H Ge X Du J Liu W Li X. Vitamin D suppresses bleomycin-induced pulmonary fibrosis by targeting the local renin-angiotensin system in the lung. Sci Rep. (2021) 11:16525. doi: 10.1038/s41598-021-96152-7 34400742 PMC8367953

[42] Wang Q Hong L Chen M Shi J Lin X Huang L. Targeting M2 macrophages alleviates airway inflammation and remodeling in asthmatic mice via mir-378a-3p/grb2 pathway. Front Mol Biosci. (2021) 8:717969. doi: 10.3389/fmolb.2021.717969 34589519 PMC8473897

[43] Zhou X Michal JJ Jiang Z Liu B. Microrna expression profiling in alveolar macrophages of indigenous chinese tongcheng pigs infected with prrsv *in vivo* . J Appl Genet. (2017) 58:539–44. doi: 10.1007/s13353-017-0410-9 28971377

[44] Huntzinger E Izaurralde E. Gene silencing by micrornas: contributions of translational repression and mrna decay. Nat Rev Genet. (2011) 12:99–110. doi: 10.1038/nrg2936 21245828

[45] Gosline SJ Gurtan AM JnBaptiste CK Bosson A Milani P Dalin S. Elucidating microrna regulatory networks using transcriptional, post-transcriptional, and histone modification measurements. Cell Rep. (2016) 14:310–9. doi: 10.1016/j.celrep.2015.12.031 PMC4831719 26748710

[46] Joshi N Walter JM Misharin AV. Alveolar macrophages. Cell Immunol. (2018) 330:86–90. doi: 10.1016/j.cellimm.2018.01.005 29370889

[47] Hou F Xiao K Tang L Xie L. Diversity of macrophages in lung homeostasis and diseases. Front Immunol. (2021) 12:753940. doi: 10.3389/fimmu.2021.753940 34630433 PMC8500393

[48] Merad M Sathe P Helft J Miller J Mortha A. The dendritic cell lineage: ontogeny and function of dendritic cells and their subsets in the steady state and the inflamed setting. Annu Rev Immunol. (2013) 31:563–604. doi: 10.1146/annurev-immunol-020711-074950 23516985 PMC3853342

[49] Bantsimba-Malanda C Marchal-Somme J Goven D Freynet O Michel L Crestani B. A role for dendritic cells in bleomycin-induced pulmonary fibrosis in mice? Am J Respir Crit Care Med. (2010) 182:385–95. doi: 10.1164/rccm.200907-1164OC 20395561

[50] Ding L Liu T Wu Z Hu B Nakashima T Ullenbruch M. Bone marrow cd11c+ Cell-derived amphiregulin promotes pulmonary fibrosis. J Immunol. (2016) 197:303–12. doi: 10.4049/jimmunol.1502479 PMC4912903 27206766

[51] Lemaire I Beaudoin H Masse S Grondin C. Alveolar macrophage stimulation of lung fibroblast growth in asbestos-induced pulmonary fibrosis. Am J Pathol. (1986) 122:205–11. PMC1888101 3946556

[52] Machiels B Dourcy M Xiao X Javaux J Mesnil C Sabatel C. A gammaherpesvirus provides protection against allergic asthma by inducing the replacement of resident alveolar macrophages with regulatory monocytes. Nat Immunol. (2017) 18:1310–20. doi: 10.1038/ni.3857 29035391

[53] Holl EK. Generation of bone marrow and fetal liver chimeric mice. Methods Mol Biol. (2013) 1032:315–21. doi: 10.1007/978-1-62703-496-8_24 PMC3947880 23943463

[54] Piguet PF Grau GE de Kossodo S. Role of granulocyte-macrophage colony-stimulating factor in pulmonary fibrosis induced in mice by bleomycin. Exp Lung Res. (1993) 19:579–87. doi: 10.3109/01902149309031729 7504622

[55] Kim YK Kim B Kim VN. Re-evaluation of the roles of drosha, export in 5, and dicer in microrna biogenesis. Proc Natl Acad Sci U.S.A. (2016) 113:E1881–9. doi: 10.1073/pnas.1602532113 PMC4822641 26976605

[56] Kolb P Upagupta C Vierhout M Ayaub E Bellaye PS Gauldie J. The importance of interventional timing in the bleomycin model of pulmonary fibrosis. Eur Respir J. (2020) 55:1–10. doi: 10.1183/13993003.01105-2019 32165401

[57] Eyal FG Hamm CR Parker JC. Reduction in alveolar macrophages attenuates acute ventilator induced lung injury in rats. Intensive Care Med. (2007) 33:1212–8. doi: 10.1007/s00134-007-0651-x 17468847

[58] Tsushima Y Jang JH Yamada Y Schwendener R Suzuki K Weder W. The depletion of donor macrophages reduces ischaemia-reperfusion injury after mouse lung transplantation. Eur J Cardiothorac Surg. (2014) 45:703–9. doi: 10.1093/ejcts/ezt489 24113322

[59] Elder A Johnston C Gelein R Finkelstein J Wang Z Notter R. Lung inflammation induced by endotoxin is enhanced in rats depleted of alveolar macrophages with aerosolized clodronate. Exp Lung Res. (2005) 31:527–46. doi: 10.1080/019021490944223 16019986

[60] Wang Y Zhang L Wu GR Zhou Q Yue H Rao LZ. Mbd2 serves as a viable target against pulmonary fibrosis by inhibiting macrophage M2 program. Sci Adv. (2021) 7:1–12. doi: 10.1126/sciadv.abb6075 PMC7775789 33277324

[61] Probst HC Lagnel J Kollias G van den Broek M. Inducible transgenic mice reveal resting dendritic cells as potent inducers of cd8+ T cell tolerance. Immunity. (2003) 18:713–20. doi: 10.1016/s1074-7613(03)00120-1 12753747

[62] Casanova-Acebes M Dalla E Leader AM LeBerichel J Nikolic J Morales BM. Tissue-resident macrophages provide a pro-tumorigenic niche to early nsclc cells. Nature. (2021) 595:578–84. doi: 10.1038/s41586-021-03651-8 PMC8923521 34135508

[63] Ural BB Yeung ST Damani-Yokota P Devlin JC de Vries M Vera-Licona P. Identification of a nerve-associated, lung-resident interstitial macrophage subset with distinct localization and immunoregulatory properties. Sci Immunol. (2020) 5:1–28. doi: 10.1126/sciimmunol.aax8756 PMC7717505 32220976

[64] Gorki AD Symmank D Zahalka S Lakovits K Hladik A Langer B. Murine ex vivo cultured alveolar macrophages provide a novel tool to study tissue-resident macrophage behavior and function. Am J Respir Cell Mol Biol. (2022) 66:64–75. doi: 10.1165/rcmb.2021-0190OC 34586974 PMC8803354

[65] Iqbal AJ McNeill E Kapellos TS Regan-Komito D Norman S Burd S. Human cd68 promoter gfp transgenic mice allow analysis of monocyte to macrophage differentiation *in vivo* . Blood. (2014) 124:e33–44. doi: 10.1182/blood-2014-04-568691 PMC4192756 25030063

[66] Oak SR Murray L Herath A Sleeman M Anderson I Joshi AD. A micro rna processing defect in rapidly progressing idiopathic pulmonary fibrosis. PloS One. (2011) 6:e21253. doi: 10.1371/journal.pone.0021253 21712985 PMC3119674

[67] Banerjee S Xie N Cui H Tan Z Yang S Icyuz M. Microrna let-7c regulates macrophage polarization. J Immunol. (2013) 190:6542–9. doi: 10.4049/jimmunol.1202496 PMC3679284 23667114

[68] Banerjee S Cui H Xie N Tan Z Yang S Icyuz M. Mir-125a-5p regulates differential activation of macrophages and inflammation. J Biol Chem. (2013) 288:35428–36. doi: 10.1074/jbc.M112.426866 PMC3853290 24151079

[69] Zhang Y Zhang M Zhong M Suo Q Lv K. Expression profiles of mirnas in polarized macrophages. Int J Mol Med. (2013) 31:797–802. doi: 10.3892/ijmm.2013.1260 23443577

[70] Xu Z Zhao L Yang X Ma S Ge Y Liu Y. Mmu-mir-125b overexpression suppresses no production in activated macrophages by targeting eef2k and ccna2. BMC Cancer. (2016) 16:252. doi: 10.1186/s12885-016-2288-z 27020049 PMC4809031

[71] Alivernini S Gremese E McSharry C Tolusso B Ferraccioli G McInnes IB. Microrna-155-at the critical interface of innate and adaptive immunity in arthritis. Front Immunol. (2017) 8:1932. doi: 10.3389/fimmu.2017.01932 29354135 PMC5760508

[72] Kurowska-Stolarska M Hasoo MK Welsh DJ Stewart L McIntyre D Morton BE. The role of microrna-155/liver X receptor pathway in experimental and idiopathic pulmonary fibrosis. J Allergy Clin Immunol. (2017) 139:1946–56. doi: 10.1016/j.jaci.2016.09.021 PMC5457127 27746237

[73] Christmann RB Wooten A Sampaio-Barros P Borges CL Carvalho CR Kairalla RA. Mir-155 in the progression of lung fibrosis in systemic sclerosis. Arthritis Res Ther. (2016) 18:155. doi: 10.1186/s13075-016-1054-6 27377409 PMC4932708

[74] Manoury B Caulet-Maugendre S Guenon I Lagente V Boichot E. Timp-1 is a key factor of fibrogenic response to bleomycin in mouse lung. Int J Immunopathol Pharmacol. (2006) 19:471–87. doi: 10.1177/039463200601900303 17026855

[75] Bobba CM Fei Q Shukla V Lee H Patel P Putman RK. Nanoparticle delivery of microrna-146a regulates mechanotransduction in lung macrophages and mitigates injury during mechanical ventilation. Nat Commun. (2021) 12:289. doi: 10.1038/s41467-020-20449-w 33436554 PMC7804938

[76] Geng S Matsushima H Okamoto T Yao Y Lu R Page K. Emergence, origin, and function of neutrophil-dendritic cell hybrids in experimentally induced inflammatory lesions in mice. Blood. (2013) 121:1690–700. doi: 10.1182/blood-2012-07-445197 PMC3591794 23305733

[77] Ashcroft T Simpson JM Timbrell V. Simple method of estimating severity of pulmonary fibrosis on a numerical scale. J Clin Pathol. (1988) 41:467–70. doi: 10.1136/jcp.41.4.467 PMC1141479 3366935

[78] Huang da W Sherman BT Lempicki RA. Systematic and integrative analysis of large gene lists using david bioinformatics resources. Nat Protoc. (2009) 4:44–57. doi: 10.1038/nprot.2008.211 19131956

