# Supplementary material for: MicroRNAs regulate alveolar macrophage homeostasis and its function in lung fibrosis
Source: Front Immunol. 2025 Jun 23;16:1598306. doi: 10.3389/fimmu.2025.1598306 (PMC12231002; doi:10.3389/fimmu.2025.1598306)
Supplement: Supplementary file 1 [file DataSheet1.docx]

Supplementary Material

# Supplemental Materials and Methods

**Genotyping**

Genotyping of *Cd11c^Cre^* and *Dicer^fl/fl^* mice was performed in adherence to the standard PCR-based procedure. The following primers were used for each mouse strain: *Cd11c^Cre^*: forward 5′-ACTTGGCAGCTGTCTCCAAG-3′ and reverse 5′-GCGAACATCTTCAGGTTCTG-3′; *Dicer^fl/fl^*: forward 5′-CCTGACAGTGACGGTCCAAAG-3′ and reverse 5′-CATGACTCTTCAACTCAAACT-3′. The floxed allele of *Dicer^fl/fl^* mice generated a 420bp PCR product while the wild type allele created a 351bp PCR product.

**Tissue preparation**

Two weeks following bleomycin injury, mice were sacrificed by CO_2_ inhalation and a standard bronchoalveolar lavage was performed. Briefly, the trachea was exposed and a small incision was made to allow catheter placement. The catheter, attached to a 1 mL syringe loaded with 1 mL of 1× PBS, was inserted 0.5 cm into the trachea, and the PBS was injected into the lungs. The solution was aspirated and re-injected for a total of 3 times, then collected. The samples were then centrifuged at 450 × g for 7 min at 4 ºC. The supernatant was frozen at -80 ºC for ELISA assay, and erythrocytes were lysed by incubation in 0.83% NH_4_Cl buffer for 4 min at room temperature.

Lungs were perfused with 1× PBS and harvested for H&E and trichome staining, hydroxyproline and protein quantification, RNA extraction, and flow cytometry. For flow cytometry, samples were minced into small pieces and incubated in PBS containing 1 mg/mL collagenase D (Roche, Basel, Switzerland), 0.01% DNase I (Worthington Biochemical Corp., Lakewood, NJ), and 3% FBS (Hyclone, San Angelo, TX) for 45 min at 37 °C, then passed through a 70 µm cell strainer. Erythrocytes were lysed by incubation in 0.83% NH_4_Cl buffer for 4 min at room temperature, then passed through a 40 µm cell strainer.

**Flow cytometry and cell sorting**

Single-cell suspensions were centrifuged at 450 × g for 7 min at 4 ºC and resuspended in ice-cold staining buffer (1× PBS containing 2% FBS). Cells were incubated with purified anti-FcγRII/III antibody (clone 2.4G2) for 15 min at 4 °C, then incubated with a mixture of fluorescent surface antibodies for 30 min at 4 °C. A full list of antibodies used can be found in the Supplemental Table 6. Stained cells were analyzed by flow cytometry using the BD FACSCelesta™ flow cytometer in coordination with the BD FACSDiva software v.8.0.2 (BD Biosciences). Data were analyzed using FlowJo software v.10.5.3 (BD Biosciences). TR-AMs (CD45^+^CD64^+^Siglec-F^hi^CD11b^lo^) and Mo-AMs (CD45^+^CD64^+^Siglec-F^lo^CD11b^hi^) from bleomycin-treated mice were sorted using the BD FACSAria™ II cell sorter in coordination with the BD FACSDiva software. The purity of the isolated populations was >95%.

**RNA extraction and quantitative RT-PCR**

Total RNA was extracted from purified AM cells using the miRNeasy Mini Kit (QIAGEN) and was reverse transcribed to cDNA using the miRCURY LNA™ RT Kit (QIAGEN). Quantitative real-time PCR reactions were prepared using miRCURY LNA SYBR Green PCR Kit (QIAGEN) and were performed using the QuantStudio 7 Flex Real-Time PCR System (Applied Biosystems). Data were collected using the QuantStudio 7 Flex Real-Time PCR System software v.1.2 (Applied Biosystems) and were analyzed using Microsoft Excel 2016 (Microsoft, Redmond, Washington). U6 primers were purchased from Exiqon (product #203907) and all other miRNA primers were purchased from QIAGEN. Relative miRNA expression was normalized to U6 expression. Mouse *Dicer1* primers: forward 5’-AGGACGACTTCCTGGAGTAT-3’ and reverse 5’-GAAGGGCGACAGAGAGATTT-3’. Mouse *Gapdh* primers: forward 5’- GGTGAAGGTCGGTGTGAACG-3’ and reverse 5’- TGTAGACCATGTAGTTGAGGTCA-3’. Relative mRNA expression was normalized to GAPDH expression.

# Supplementary Figures

#
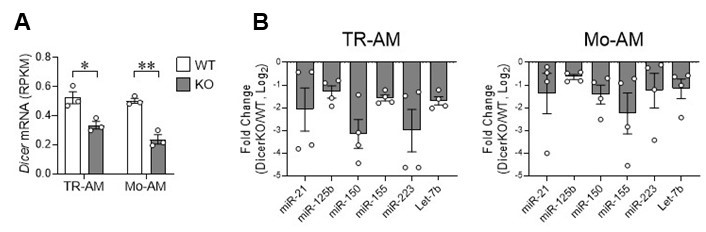


**Supplemental Figure 1.** **Deletion of Dicer and miRNAs in AMs from CD11c-DicerKO mice.** ( (A) mRNA expression of *Dicer* in TR-AMs and Mo-AMs from bleomycin (BLM)-treated WT and DicerKO mice analyzed by bulk RNA-seq. (B) Representative miRNA expression in TR-AMs and Mo-AMs from BLM-treated WT and Dicer KO mice analyzed by qRT-PCR. Bars represent mean ± SEM of biologically independent samples. All *P* values were obtained by the Student’s two-tailed unpaired *t* test. **P* < 0.05. ***P* < 0.01.


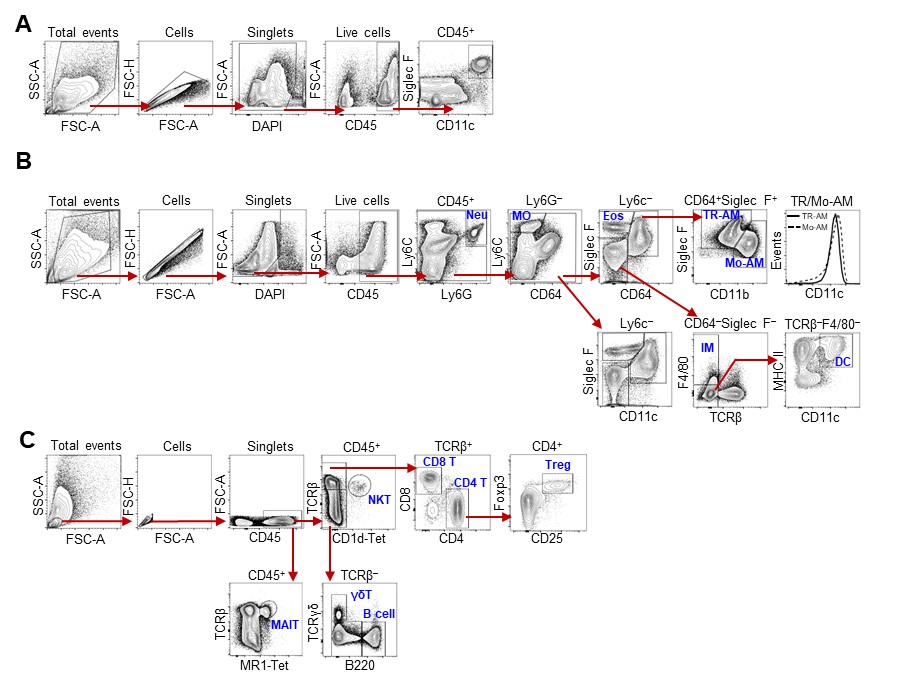


**Supplemental Figure 2.** **Flow cytometry gating strategy for immune cells in BAL and lungs.**  (A) Lung single-cell suspensions were prepared from *Dicer^l/fl^* adult naïve mice. After excluding debris (FSC-A vs. SSC-A) and doublets (FSC-A vs. FSC-H), and dead cells (DAPI^+^), CD11c and Siglec F expression was analyzed for TR-AMs (CD11c^hi^Siglec F^hi^) within CD45^+^ cells. (B) Gating strategy for myeloid cells analyzed from BAL or lungs of saline- or BLM-treated mice, including neutrophils (Neu, Ly6G^+^Ly6C^+^), monocytes (MO, Ly6G^‒^Ly6C^+^CD64^‒^), eosinophils (Eos, Ly6G^‒^Ly6C^‒^CD64^‒^Siglec-F^+^), TR-AMs (CD64^+^Siglec-F^hi^CD11b^lo^), Mo-AMs (CD64^+^Siglec-F^lo^CD11b^hi^), interstitial macrophages (IM, CD64^‒^Siglec-F^‒^F4/80^+^), and dendritic cells (DCs, CD64^‒^Siglec-F^‒^F4/80^‒^CD11c^+^MHC-II^+^). (C) Gating strategy for lymphocytes analyzed from BAL or lungs of saline- or BLM-treated mice, including CD4 T (TCRβ^+^CD4^+^), CD8 T (TCRβ^+^CD8^+^), Treg (TCRβ^+^CD4^+^CD25^+^Foxp3^+^), NKT (TCRβ^+^CD1d-tetramer^+^), MAIT (TCRβ^+^MR1-tetramer^+^), γδ T (TCRβ^‒^TCRγδ^+^) and B cells (TCRβ^‒^TCRγδ^‒^B220^+^).


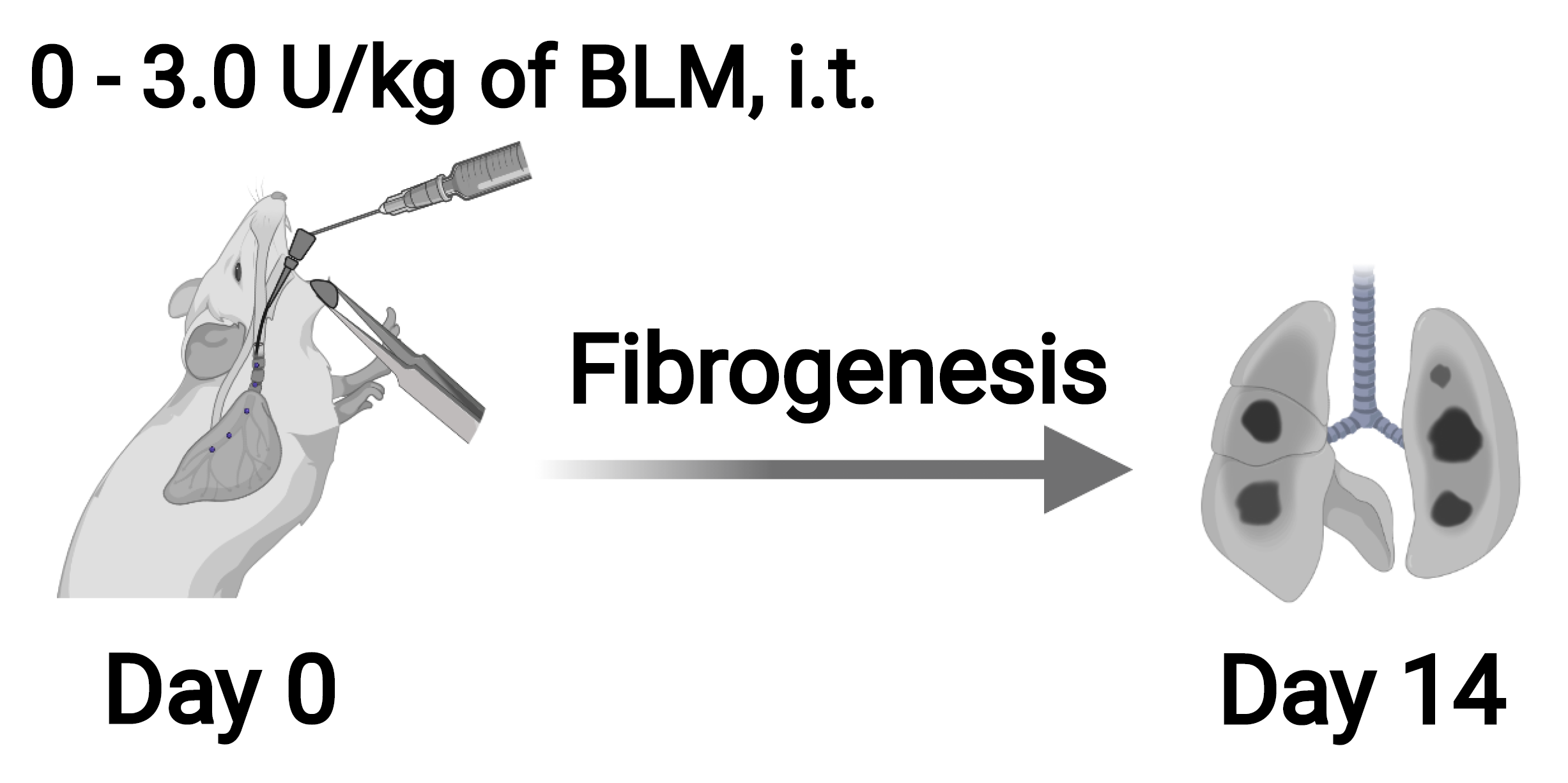


**A**

**B**

**C**

**C57BL/6**

**Supplemental Figure 3. Optimization of bleomycin-induced pulmonary fibrosis mouse model.** (**A**) Schematic representation of the BLM-induced lung fibrosis model. (**B**) Survival rates of C57BL/6 mice given the indicated doses of BLM or saline control for 2 weeks after treatment. (**C**) Mouse lungs were collected to assess hydroxyproline levels at day 14 after BLM injury. Bars represent mean ± SEM of biologically independent samples. All *P* values were obtained by the Student’s two-tailed unpaired *t* test for comparisons between each BLM group and saline control group. **P* < 0.05. ***P* < 0.01. *** *P* < 0.001.


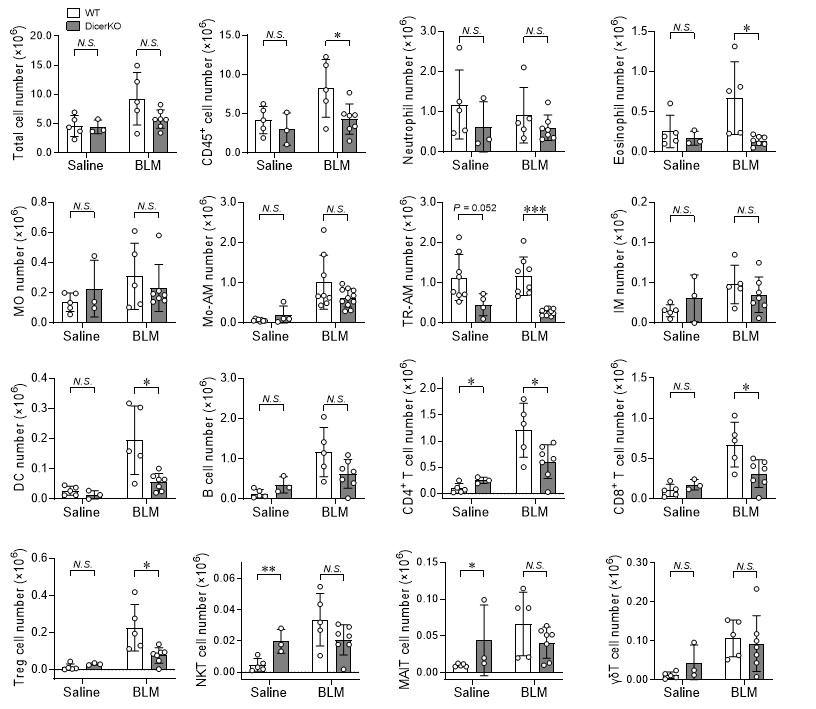
 **Supplemental Figure 4**. **Lung immunoprofiles in the saline- or BLM-treated WT and CD11c-DicerKO mice.** *Dicer^fl/fl^* (WT) and *Cd11c^cre^Dicer^fl/fl^* (KO) mice were intratracheally administered with saline or BLM and the lungs were harvested for flow cytometry analysis at day 15 after treatment. Data shown are the absolute numbers of total lung cells and the indicated immune cell populations. Each dot represents one mouse and bars represent mean ± SD of biologically independent samples in each panel. All *P* values obtained by the Student’s two-tailed unpaired *t* test. **P* < 0.05; ***P* < 0.01; ****P* < 0.001. *N.S.*, not significant.

**
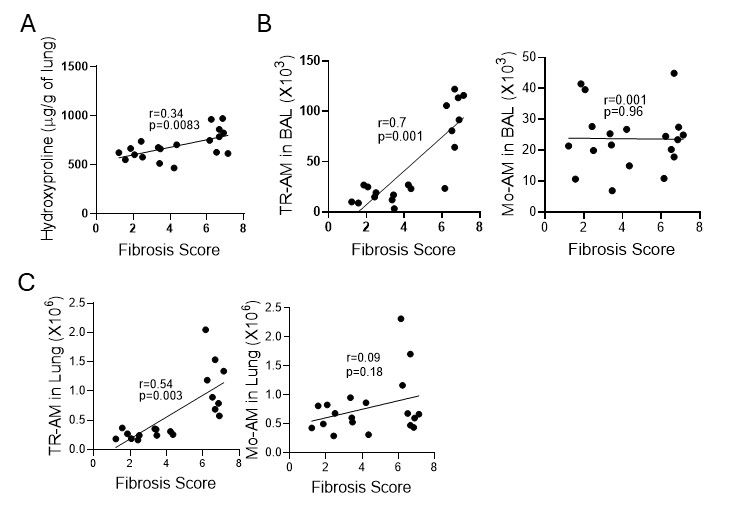
**

**Supplemental Figure 5**. **Validating the relationship between histology microphotographs–based BLM induced lung fibrosis scores and hydroxyproline levels, including lung and BAL fluid containing TR-AM and Mo-AM populations.** Data shown are the relative correlation of Ashcroft fibrosis score compared with hydroxyproline levels **(A),** numbers of total TR-AM and Mo-AM in BAL **(B**), and TR-AM and Mo-AM in lung **(C)** after bleomycin induction**.** Each dot represents one mouse, R represent regression coefficient, and *P* represents the significance after regression analysis **P* < 0.05; ***P* < 0.01; ****P* < 0.001 and *P* >0.05 is *N.S.*, not significant.
